# Development and transdifferentiation into inner hair cells require Tbx2

**DOI:** 10.1093/nsr/nwac156

**Published:** 2022-08-09

**Authors:** Zhenghong Bi, Xiang Li, Minhui Ren, Yunpeng Gu, Tong Zhu, Shuting Li, Guangqin Wang, Suhong Sun, Yuwei Sun, Zhiyong Liu

**Affiliations:** Institute of Neuroscience, State Key Laboratory of Neuroscience, CAS Center for Excellence in Brain Science and Intelligence Technology, Chinese Academy of Sciences, Shanghai 200031, China; Institute of Neuroscience, State Key Laboratory of Neuroscience, CAS Center for Excellence in Brain Science and Intelligence Technology, Chinese Academy of Sciences, Shanghai 200031, China; Institute of Neuroscience, State Key Laboratory of Neuroscience, CAS Center for Excellence in Brain Science and Intelligence Technology, Chinese Academy of Sciences, Shanghai 200031, China; University of Chinese Academy of Sciences, Beijing 100049, China; Institute of Neuroscience, State Key Laboratory of Neuroscience, CAS Center for Excellence in Brain Science and Intelligence Technology, Chinese Academy of Sciences, Shanghai 200031, China; University of Chinese Academy of Sciences, Beijing 100049, China; Institute of Neuroscience, State Key Laboratory of Neuroscience, CAS Center for Excellence in Brain Science and Intelligence Technology, Chinese Academy of Sciences, Shanghai 200031, China; Institute of Neuroscience, State Key Laboratory of Neuroscience, CAS Center for Excellence in Brain Science and Intelligence Technology, Chinese Academy of Sciences, Shanghai 200031, China; University of Chinese Academy of Sciences, Beijing 100049, China; Institute of Neuroscience, State Key Laboratory of Neuroscience, CAS Center for Excellence in Brain Science and Intelligence Technology, Chinese Academy of Sciences, Shanghai 200031, China; University of Chinese Academy of Sciences, Beijing 100049, China; Institute of Neuroscience, State Key Laboratory of Neuroscience, CAS Center for Excellence in Brain Science and Intelligence Technology, Chinese Academy of Sciences, Shanghai 200031, China; University of Chinese Academy of Sciences, Beijing 100049, China; Institute of Neuroscience, State Key Laboratory of Neuroscience, CAS Center for Excellence in Brain Science and Intelligence Technology, Chinese Academy of Sciences, Shanghai 200031, China; University of Chinese Academy of Sciences, Beijing 100049, China; Institute of Neuroscience, State Key Laboratory of Neuroscience, CAS Center for Excellence in Brain Science and Intelligence Technology, Chinese Academy of Sciences, Shanghai 200031, China; University of Chinese Academy of Sciences, Beijing 100049, China; Shanghai Center for Brain Science and Brain-Inspired Intelligence Technology, Shanghai 201210, China

**Keywords:** Tbx2, cochlea, inner hair cell, supporting cell, Ikzf2, outer hair cell

## Abstract

Atoh1 is essential for the development of both outer hair cells (OHCs) and inner hair cells (IHCs) in the mammalian cochlea. Whereas *Ikzf2* is necessary for OHC development, the key gene required for IHC development remains unknown. We found that deletion of Tbx2 in neonatal IHCs led to their transdifferentiation into OHCs by repressing 26.7% of IHC genes and inducing 56.3% of OHC genes, including *Ikzf2*. More importantly, persistent expression of Tbx2 coupled with transient Atoh1 expression effectively reprogrammed non-sensory supporting cells into new IHCs expressing the functional IHC marker vGlut3. The differentiation status of these new IHCs was considerably more advanced than that previously reported. Thus, Tbx2 is essential for IHC development and co-upregulation of Tbx2 with Atoh1 in supporting cells represents a new approach for treating deafness related to IHC degeneration.

## Teaser

How to produce the inner hair cells?

## INTRODUCTION

Precise specification of distinct cell fates is crucial for organogenesis and to define the molecular mechanisms underlying this fundamental developmental event, a fascinating model that has been widely used is the mouse cochlea. In the cochlea, two subtypes of sound receptor cells—the inner hair cells (IHCs) and the outer hair cells (OHCs)—are located in the auditory epithelium, which is also referred to as the organ of Corti [1–3]. IHCs and OHCs are derived from the same progenitors expressing Atoh1, a key b-HLH transcription factor (TF) necessary for generating both IHCs and OHCs [4,5]. Adjacent to HCs are distinct subtypes of non-sensory cochlear supporting cells (SCs) that are arranged from the medial to lateral portion and named inner border cells (IBCs), inner phalangeal cells (IPhs), pillar cells (PCs) and Deiters’ cells (DCs) [1,2,6]. Whereas the cochlear SCs of non-mammalian vertebrates, including birds and fish, can regenerate HCs upon damage, the SCs in mammals lack this regenerative capacity [7,8]. Therefore, damage or degeneration of either OHCs or IHCs results in permanent hearing impairment in mammals, including humans.

The IHCs and OHCs share various pan-HC markers such as *Myo6* and *Myo7a*, but the cells also differ in several aspects. OHCs are sound amplifiers and specifically express Prestin, a motor protein encoded by *Slc26a5* [9,10] and *Slc26a5^–^^/^^–^* mice display severe hearing impairment [11]. Conversely, IHCs are primary sensory cells and specifically express vGlut3 (encoded by *Slc17a8*), Otoferlin and Slc7a14 [12–15]. The *Slc17a8^–^^/^^–^* and *Otoferlin^–^^/^^–^* mice exhibit profound deafness because both vGlut3 and Otoferlin are heavily involved in the packaging and exocytosis of ribbon synapse vesicles containing the excitatory neurotransmitter glutamate in the IHCs, respectively. Recently, *Insm1* and *Ikzf2* were reported as key regulators of OHC development and, accordingly, OHCs tend to transdifferentiate into IHCs or IHC-like cells in *Insm1* or *Ikzf2* mutant mice [16,17].

In contrast to the aforementioned identification of genes for OHC development, it remains poorly understood what gene is needed for normal IHC development as well as which gene, together with *Atoh1*, can transdifferentiate cochlear SCs into the vGlut3+ IHCs. Addressing this question would undoubtedly provide new insights into the molecular mechanisms underlying IHC development and regeneration. In this study, we found that T-box transcription factor 2 (*Tbx2*) is highly and persistently expressed in IHCs but not OHCs. When *Tbx2* is conditionally deleted in neonatal ages, most if not all IHCs tend to transdifferentiate into OHCs, with upregulation and downregulation of 56.3% and 26.7% of OHC and IHC genes, respectively. Moreover, IHC fate also becomes unstable when *Tbx2* is conditionally ablated in adult IHCs. To the best of our knowledge, *Tbx2* is the first gene reported to be necessary in IHC development. Besides revealing the critical role of *Tbx2* in normal IHC development, we demonstrate that *Tbx2* and *Atoh1* together are sufficient to transform neonatal cochlear IBCs/IPhs into vGlut3+ new IHCs, with a reprogramming efficiency of ∼29.5%. The new IHCs also express the other IHC markers Otoferlin and Slc7a14 and harbor bird-wing-like stereocilia. Collectively, our findings uncover the essential role of Tbx2 in preventing differentiating and mature IHCs from developing into OHCs. Our study further shows that Tbx2, together with Atoh1, promotes transdifferentiation of neonatal IBCs/IPhs into IHCs expressing vGlut3, with the endogenous cochlear IHCs remaining intact. This experimental paradigm should facilitate future IHC regeneration and highlight a new strategy of treating IHC degeneration-associated deafness in clinic.

## RESULTS

### *Tbx2* is highly expressed in IHCs but not OHCs

**Figure 1.**
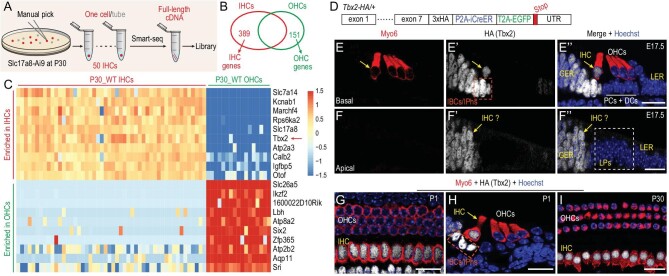
Tbx2 is highly expressed in IHCs but not OHCs. (A) and (B) Illustration of single-cell RNA-seq of adult IHCs at P30 by using smart-seq (A); 389 IHC genes and 151 OHC genes are identified (B). (C) Heat map showing top examples of differently expressed genes between IHCs and OHCs at P30. (D) Diagram of Tbx2*3 × HA-P2A-iCreER-T2A-EGFP/+ (*Tbx2*-HA/+); for details, please refer to Supplementary Fig. S1A–F. (E)–(I) Dual staining of HC marker Myo6 and HA (Tbx2) in cochlear samples from *Tbx2*-HA/+ mice at E17.5 (E)–(F’’), P1 (G) and (H) and P30 (I). Yellow arrows in (E)–(F’’): IHCs; dotted squares in (E’) and (H): IBCs/IPhs. IHCs, inner hair cells; OHCs, outer hair cells; GER, greater epithelial ridge cells; LER, lesser epithelial ridge cells; LPs, lateral progenitors; IBCs/IPhs, inner border cells/inner phalangeal cells; PCs, pillar cells; DCs, Deiters’ cells. Scale bar: 20 μm.

We aimed to identify genes that are highly expressed in IHCs but are depleted in OHCs; our hypothesis was that these genes are the key candidate regulators in IHC development. First, *Slc17a8^iCreER/^*^+^; *Rosa26*-CAG-LSL-Tdtomato (Ai9)/+ (abbreviated as Slc17a8-Ai9) mice were administered tamoxifen (TMX) at Postnatal Day 2 (P2) and P3 [18], before the cochlear sensory epithelium was dissected, digested and dissociated at P30. Only the IHCs in the tissue were Tdtomato+, and 50 Tdtomato+ endogenous IHCs at P30 (P30_WT IHCs) were manually picked and subject to single-cell RNA-seq by using the smart-seq approach (Fig. 1A). Comparison of gene-expression profiles between these 50 P30_WT IHCs and 17 wild-type (WT) OHCs at P30 (P30_WT OHCs) we reported previously [19] revealed 2162 differentially expressed genes (DEGs) [absolute value of fold-change (FC) in gene expression: >4; *P* < 0.05] (Supplementary Table S1). By using a transcripts per million (TPM) value of >16 as the selection criterion [19], 389 and 151 genes were respectively defined as IHC and OHC genes (Fig. 1B and Supplementary Table S1). These genes included the known IHC markers *Slc17a8, Tbx2, Otof* and *Slc7a14* [12–15,17] and the OHC markers *Slc26a5, Ikzf2, Lbh* and *Sri* [10,16,20,21] (Fig. 1C). Notably, *Tbx2* was found to be the top TF gene highly expressed in IHCs but not OHCs (red arrow in Fig. 1C). However, the Tbx2 protein expression pattern in the cochlea remains poorly characterized.

Next, we generated a new knockin mouse strain, Tbx2*3 × HA-P2A-iCreER-T2A-EGFP/+ (abbreviated as *Tbx2*-HA/+), wherein three HA tags were fused to the C-terminus of Tbx2 (Fig. 1D and Supplementary Fig. S1A–F) and the Tbx2 expression pattern was primarily characterized using an anti-HA antibody. In agreement with previous reports [22,23], the HA (Tbx2) expression domain was found to overlap with that of Sox2 in the otocyst, but not in the hindbrain, at Embryonic Day 9.5 (E9.5) (Supplementary Fig. S1G–G’’). Tbx2 was broadly expressed in cochlear duct cells at E13.5. However, at E15.5, although Tbx2 expression was maintained in the medial portion including Myo6+ IHCs, the expression was undetectable in lateral progenitors (LPs) that eventually become OHCs, PCs and DCs in the basal turn (Supplementary Fig. S1H–H’’). By contrast, Tbx2 continued to be broadly expressed in the apical turn at E15.5 (Supplementary Fig. S1I–I’’). The wave of Tbx2 downregulation was largely completed by E17.5, when Tbx2 was undetectable in basal OHCs, PCs and DCs, and apical LPs (Fig. 1E–F’’). As expected, Tbx2 was highly expressed in IHCs, but not OHCs, at P1 (Fig. 1G and H), P15 and P30 (Fig. 1I). Collectively, these results showed that Tbx2 is persistently and specifically expressed in both differentiating and mature IHCs, prompting us to hypothesize that Tbx2 is essential for IHC development.

### Neonatal IHCs transdifferentiate into OHCs when *Tbx2* is conditionally deleted

Our aforementioned hypothesis was supported by genetic evidence obtained from two distinct *Tbx2* mutant strains. The first was a germ-line *Tbx2* knockout mouse strain (*Tbx2^+/^^–^*) in which the entire *Tbx2* locus (∼9.6 kbp) was deleted (Supplementary Fig. S2A–C). *Tbx2^–/–^* mice die at early embryonic ages due to cardiac defect [24]. Importantly, the OHC-specific marker Prestin was weakly detected in *Tbx2^+/^^–^* but not *Tbx2^+/+^* IHCs at P7, P14 and P42. Because the IHC phenotypes in *Tbx2^+/^^–^* were mild, detailed analyses are not presented and we focused our study on the second mutant strain that we generated: *Tbx2^flox/+^*, wherein the second exon of *Tbx2* was flanked by two loxp sequences (Supplementary Fig. S2D–I). Here, WT mice (control group) and *Slc17a8^iCreER/^*^+^; *Tbx2^flox/^^–^* conditional knockout mice (abbreviated as Tbx2 cko mice; experimental group) were administered TMX at P2 and P3 and examined at different ages.

**Figure 2.**
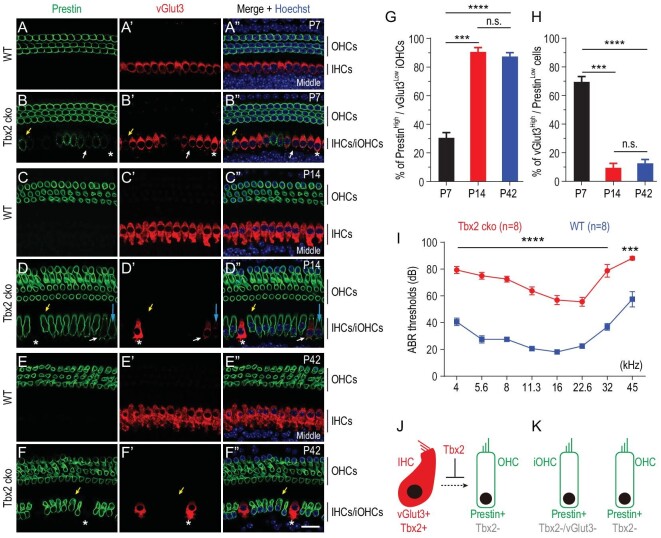
Loss of Tbx2 causes IHCs to gradually decrease vGlut3 but increase Prestin expression. (A)–(F’’) Dual whole-mount staining of vGlut3 and Prestin in cochlear samples from wild-type mice [WT; (A)–(A’’), (C)–(C’’) and (E)–(E’’)] and *Tbx2* conditional knockout mice [Tbx2 cko; (B)–(B’’), (D)–(D’’) and (F)–(F’’)] at P7, P14 and P42, respectively. Yellow arrows: one Prestin^High^/vGlut3^Low^ iOHC; white arrows: one vGlut3^High^/Prestin^Low^ iOHC. Asterisks: IHCs that did not undergo cell-fate conversion; to simplify analysis, these cells were included in vGlut3^High^/Prestin^Low^ population. (G) and (H) Quantification of Prestin^High^/vGlut3^Low^ iOHCs (G) and vGlut3^High^/Prestin^Low^ cells (H) in Tbx2 cko mice at P7, P14 and P42. Data are presented as means ± SEM. ***P < 0.001; ****P < 0.0001; n.s.: no significant difference. (I) ABR measurements between WT (blue line) and Tbx2 cko (red line) mice at P42; significantly different at all frequencies: ***P < 0.001; ****P < 0.0001. (J) and (K) Simplified model highlighting role of Tbx2 in IHC fate stabilization. After Tbx2 is deleted, IHCs become iOHCs (K). Scale bar: 20 μm.

At P7, IHCs were vGlut3+/Prestin– in WT mice (Fig. 2A–A’’), whereas ectopic Prestin expression at heterogeneous levels was detected in IHCs in Tbx2 cko mice (arrows in Fig. 2B–B’’). Notably, relative to Prestin– IHCs (asterisks in Fig. 2B–B’’), Prestin+ IHCs showed different extents of diminished vGlut3 expression. At P14, Prestin remained specifically expressed in WT OHCs (Fig. 2C–C’’), but the Prestin level in Tbx2 cko IHCs was further increased and became comparable to that in endogenous OHCs (Fig. 2D–D’’). Conversely, vGlut3 expression remained heterogeneous, either being detected at low levels (blue arrows in Fig. 2D–D’’) or becoming undetectable (yellow arrows in Fig. 2D–D’’). Moreover, the Prestin level was homogenous in the majority of Prestin+ IHCs. This suggested that the upregulation of Prestin expression was more rapid than the downregulation of vGlut3 expression by P14. As expected, the vGlut3 level in Prestin– IHCs (asterisks in Fig. 2D–D’’) was as high as that in WT IHCs (Fig. 2C–C’’).

At P42, relative to the expression in WT IHCs (Fig. 2E–E’’), vGlut3 expression was undetectable in all Prestin+ IHCs (yellow arrows in Fig. 2F–F’’) but remained intact in Prestin– IHCs (asterisks in Fig. 2F–F’’). Thus, our data supported the notion that IHCs tend to transdifferentiate into OHCs when *Tbx2* is absent. Hereafter, to differentiate between the IHCs that did or did not undergo cell-fate change (asterisks in Fig. 2), the Prestin+ IHCs were defined as induced-OHCs derived from IHCs (abbreviated as iOHCs) in which vGlut3 expression was either decreased or completely lost.

### Cell-fate conversion of iOHCs is largely completed by P14

We next estimated the progression of cell-fate conversion in iOHCs between P7 and P42. First, we quantified the cells in entire cochlear turns that expressed high levels of Prestin (Prestin^High^) but low (or undetectable) levels of vGlut3 (vGlut3^Low^). The percentage of these cells—defined as Prestin^High^/vGlut3^Low^ iOHCs (yellow arrows in Fig. 2B-B’’, yellow and blue arrows in Fig. 2D–D’’, and yellow arrows in Fig. 2F–F’’)—was the lowest (30.5% ± 3.7%) at P7 (*n* = 3) and was significantly increased to 90.5% ± 3.1% at P14 (*n* = 3) and 87.4% ± 2.7% at P42 (*n* = 3) (Fig. 2G). Although a Tbx2 antibody was not available for validating the absence of Tbx2, the Prestin^High^/vGlut3^Low^ iOHCs are likely to correspond to the endogenous IHCs in which *Tbx2* was successfully deleted. Second, we quantified cells that expressed high levels of vGlut3 (vGlut3^High^) but low (or undetectable) levels of Prestin (Prestin^Low^); the percentage of these cells—defined as vGlut3^High^/Prestin^Low^ cells—was the highest (69.5% ± 3.7%) at P7 and was drastically decreased to 9.5% ± 3.1% at P14 and 12.6% ± 2.7% at P42 (Fig. 2H).

Notably, we expected the vGlut3^High^/Prestin^Low^ cells to include two subpopulations: (i) the iOHCs that were in the early process of cell-fate conversion (white arrows in Fig. 2B–B’’ and Fig. 2D–D’’); and (ii) the endogenous IHCs that did not undergo cell-fate change (asterisks in Fig. 2), either due to their lack of responsiveness to Tbx2 deletion or due to unsuccessful deletion of Tbx2. Thus, our results suggested that although the cell-fate conversion rate was not synchronized in distinct iOHCs, the conversion was largely completed at P14, because no significant difference was observed between P14 and P42 (Fig. 2G and H). Because vGlut3^High^/Prestin^Low^ cells accounted for only ∼12.6% of the cells in the IHC region at P42 (Fig. 2H), the hearing thresholds of Tbx2 cko mice (*n* = 8) at all tested frequencies were significantly higher than those of WT mice (*n* = 8) (Fig. 2I).

We performed double-labeling for Prestin with two additional IHC markers—Otoferlin (Supplementary Fig. S3A–B’’) and Slc7a14 (Supplementary Fig. S3C–D’’)—at P42. Briefly, Otoferlin and Slc7a14 exhibited similar expression patterns to vGlut3 (Supplementary Fig. S3E and F). Moreover, relative to WT IHCs (white circles in Supplementary Fig. S3G–G’’), iOHCs harbored fewer ribbon synapses (yellow circles in Supplementary Fig. S3H–H’’ and Supplementary Fig. S3I). Considering these results collectively, we propose that Tbx2 plays an essential role in IHC development by repressing the expression of OHC genes or preventing the transdifferentiation of IHCs into OHCs (Fig. 2J); thus, following the loss of Tbx2, neonatal IHCs gradually differentiate into iOHCs (Fig. 2K).

### IHC genes are globally repressed but OHC genes are derepressed in iOHCs

**Figure 3.**
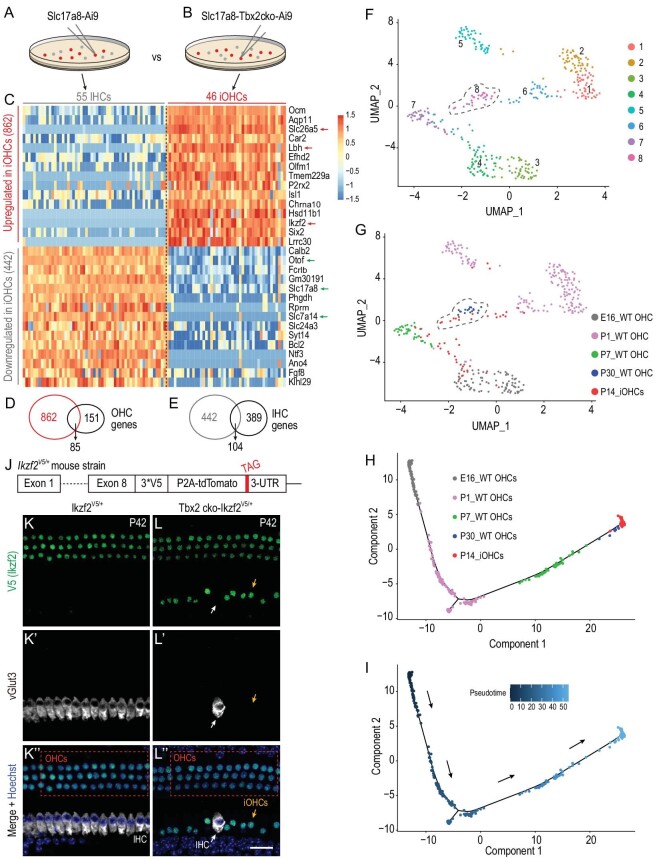
Single-cell transcriptomic profiling of iOHCs. (A) and (B) Manual picking of P14_WT IHCs from Slc17a8-Ai9 mice (A) and P14_iOHCs from Slc17a8-Tbx2cko-Ai9 mice (B). (C) Transcriptomic comparison between P14_WT IHCs and P14_iOHCs. Relative to their expression in P14_WT IHCs, 862 and 442 genes are significantly upregulated and downregulated in P14_iOHCs, respectively. (D) and (E) Overlapping genes: 85 genes, including *Slc26a5, Lbh* and *Ikzf2* (red arrows in C), overlap between the 862 upregulated and 151 defined OHC genes (D); and 104 genes, including *Otof, Slc17a8* and *Slc7a14* [green arrows in (C)], overlap between the 442 downregulated and 389 defined IHC genes. (F) and (G) UMAP analysis of cell mixtures covering E16_WT OHCs, P1_WT OHCs, P7_WT OHCs, P30_WT OHCs and P14_iOHCs; eight main cell clusters are revealed (F). Notably, 7/46 (15.2%) P14_iOHCs are assigned to Cluster 8 (gray dotted circle), which primarily comprises P30_WT OHCs. (H) and (I) Trajectory analysis of same cell mixtures as in (F) and (G). Arrows in (I): calculated developmental ages. (J) Simple illustration of how the *Ikzf2^V5/+^* mouse strain is constructed. Briefly, three V5 tags are fused to Ikzf2 C-terminus; please also refer to Supplementary Fig. S5 for details. (K)–(L’’) Dual staining of V5 (Ikzf2) and vGlut3 in control *Ikzf2^V5/+^*(K)–(K’’) and Tbx2 cko -*Ikzf2^V5/+^*(L)–(L’’) cochleae at P42. Yellow arrows in (L)–(L’’): one iOHC expressing V5 (Ikzf2) but not vGlut3; white arrows in (L)–(L’’): one endogenous IHC not expressing V5 (Ikzf2) and maintaining vGlut3 expression. Scale bar: 20 μm.

Next, we monitored the degree of cell-fate change by single-cell transcriptomic analysis. We comprehensively characterized the transcriptomic profiles of 55 WT IHCs at P14 (P14_WT IHCs) from Slc17a8-Ai9 mice and 46 iOHCs at P14 (P14_iOHCs) from *Slc17a8^iCreER/^*^+^; *Tbx2^flox/^^–^*; Ai9/+ mice (abbreviated as Slc17a8-Tbx2cko-Ai9 mice) (Fig. 3A and B). Relative to the expression in P14_WT IHCs, 862 and 442 genes were significantly (absolute value of FC > 4; *P* < 0.05) upregulated and downregulated, respectively, in P14_iOHCs (Fig. 3C and Supplementary Fig. S4A). All identified DEGs are included in Supplementary Table S2. We found that 56.3% (85/151) of the OHC genes were drastically increased and 26.7% (104/389) of the IHC genes were markedly decreased in the P14_iOHCs (Fig. 3D and E) and, notably, the upregulated OHC genes included *Slc26a5, Lbh* and *Ikzf2* (red arrows in Fig. 3C) and the downregulated IHC genes included *Otof, Slc17a8* and *Slc7a14* (green arrows in Fig. 3C).

To determine the differentiation status of endogenous WT OHCs that was most similar to that of P14_iOHCs, we pooled the 46 P14_iOHCs from this study with the 87 WT OHCs at E16 (E16_WT OHCs), 170 WT OHCs at P1 (P1_WT OHCs) and 39 WT OHCs at P7 (P7_WT OHCs) from one previous single-cell RNA-seq study [25] and the 17 P30_WT OHCs from another study [19]. In total, eight clusters were revealed through uniform manifold approximation and projection (UMAP) analysis (Fig. 3F). Notably, 7/46 (15.2%) of the P14_iOHCs belonged to Cluster 8, which mainly included the P30_WT OHCs, whereas the remaining 39/46 (84.8%) were assigned to other clusters (Fig. 3G). Moreover, trajectory analysis by using Monocle revealed that P14_iOHCs aggregated with P30_WT OHCs (Fig. 3H and I), supporting the notion that P14_iOHCs are more similar to P30_WT OHCs than to WT OHCs at other ages. A similar pattern was obtained when we included additional IHCs obtained from the same single-cell RNA-seq study (Supplementary Fig. S4B and C). Together, these results suggested that upon Tbx2 loss, the IHCs became iOHCs that were most closely related to P30_WT OHCs.

### Ikzf2 protein is also expressed in iOHCs

The similarity between iOHCs and endogenous OHCs led us to predict that Ikzf2 protein (also known as Helios), a key regulator of OHC development [16,19], would be expressed in iOHCs. Because a suitable commercial Ikzf2 antibody for immunostaining was unavailable, we constructed a new knockin mouse strain, Ikzf2*3 × V5-P2A-Tdtomato/+ (abbreviated as *Ikzf2^V5/+^*), wherein three V5 tags were fused to the C-terminus of Ikzf2 (Fig. 3J and Supplementary Fig. S5A–C). The correct gene targeting of *Ikzf2^V5/+^*was confirmed through Southern blotting and tail-DNA PCR (Supplementary Fig. S5D and E). Thus, a V5 antibody could be used to detect Ikzf2 protein. Here, we did not use Tdtomato as a reporter of *Ikzf2* mRNA expression because the signal-to-noise ratio of Tdtomato was lower than that of the V5 antibody.

Co-staining of V5 (Ikzf2) and vGlut3 showed that OHCs (dotted square in Fig. 3K’’), but not IHCs, expressed Ikzf2 in *Ikzf2^V5/+^* mice at P42 (Fig. 3K–K’’). By contrast, in *Slc17a8^iCreER/^*^+^; *Tbx2^flox/^^–^; Ikzf2^V5/+^* (Tbx2cko-Ikzf2^V5/+^) mice at P42, Ikzf2 was expressed, besides in OHCs (dotted square in Fig. 3L’’), in an additional row of cells including iOHCs (yellow arrows in Fig. 3L–L’’), but not in IHCs that did not change cell fate and accordingly maintained vGlut3 expression (white arrows in Fig. 3L–L’’). This agreed with the marked *Ikzf2* mRNA upregulation in the iOHCs (Fig. 3C and Supplementary Fig. S4A). Altogether, these results showed that both *Ikzf2* mRNA and protein were expressed in iOHCs. Because ectopic Ikzf2 is known to be sufficient for promoting IHC transdifferentiation into OHCs [16,19], the detection here of Ikzf2 protein further strengthened the ‘OHC’ features of the iOHCs.

### Tbx2 is necessary for maintaining cell fate of adult cochlear IHCs

**Figure 4.**
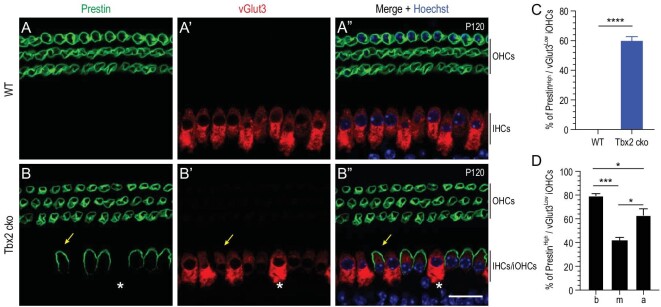
Adult IHCs also transdifferentiate into OHCs when Tbx2 is conditionally deleted. (A)–(B’’) Double staining of Prestin and vGlut3 in WT (A)–(A’’) and Tbx2 cko (B)–(B’’) cochleae. Yellow arrows in (B)–(B’’): one iOHC with high Prestin expression but low vGlut3 expression; white asterisks: one suspected endogenous IHC maintaining high vGlut3 expression and not expressing Prestin. (C) Averaged percentage of Prestin^High^/vGlut3^Low^ iOHCs in all turns of Tbx2 cko cochleae. Data are presented as means ± SEM. ****P < 0.0001. No iOHC is detected in WT cochleae. (D) Percentages of Prestin^High^/vGlut3^Low^ iOHCs in basal (b), middle (m) and apical (a) turns of Tbx2 cko cochleae. Data are presented as means ± SEM. *P < 0.05; ***P < 0.001. Scale bar: 20 μm.

We next investigated whether Tbx2 is also required for maintaining the cell fate of fully mature IHCs at adult ages. WT (*n* = 3) and Tbx2 cko (*n* = 3) mice were administered TMX at P60 and P61 and then analysed at P120. Three rows of Prestin+ OHCs and one row of vGlut3+ IHCs were well aligned in WT cochleae (Fig. 4A–A’’), whereas additional but discontinuous Prestin^High^/vGlut3^Low^ cells existed in the IHC region of Tbx2 cko cochleae (yellow arrows in Fig. 4B–B’’). According to our aforementioned criterion, Prestin^High^/vGlut3^Low^ cells were defined as iOHCs, and the nearby vGlut3^High^/Prestin^Low^ IHCs were suspected of being endogenous IHCs in which Tbx2 was not successfully deleted (asterisks in Fig. 4B–B’’). When we grouped the iOHCs in all the cochlear turns together and quantified the results, the calculated percentage of Prestin^High^/vGlut3^Low^ iOHCs at P120 was 60.0% ± 2.7% (Fig. 4C). Moreover, the middle turn was found to contain the fewest and the basal turn the most iOHCs when the cells in the three turns were counted separately (Fig. 4D).

Notably, vGlut3 was undetectable in iOHCs at P42 when *Tbx2* was deleted at P2 and P3 (arrows in Fig. 2F–F’’), but vGlut3 was detectable, albeit faintly, in iOHCs at P120 (2 months after *Tbx2* deletion at P60/P61) (arrows in Fig. 4B–B’’). Collectively, our data support the view that Tbx2 is required in maintaining or stabilizing the IHC fate at both neonatal (P2/P3) and adult (P60/P61) ages. In the absence of Tbx2, IHCs tend to transdifferentiate into OHCs and become iOHCs. However, the cell-fate conversion rate might be lower at adult ages since fewer IHCs transdifferentiated into iOHCs in adult (Fig. 4C) than neonatal (Fig. 2A–G) stages.

### A new genetic model to induce temporal Atoh1 but permanent Tbx2 expression

**Figure 5.**
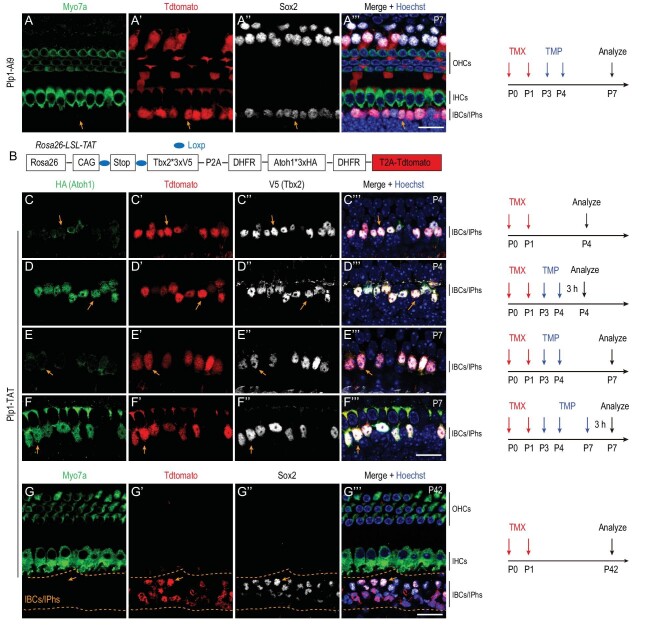
Successful transient Atoh1 and permanent Tbx2 ectopic expression in Plp1-TAT mice. (A)–(A’’’) Triple labeling for Myo7a, Tdtomato and Sox2 in control cochleae of Plp1-Ai9 mice first administered TMX (red arrows) and TMP (blue arrows) and then analysed at P7 (as depicted on the right). Orange arrows in (A)–(A’’’): one IBC/IPh that is Tdtomato+/Sox2+/Myo7a–. (B) Simple cartoon illustrating genetic model used to conditionally induce transient Atoh1 and permanent Tbx2 expression; please also refer to Supplementary Fig. S6 for details. (C)–(F’’’) Triple labeling for HA (Atoh1), Tdtomato and V5 (Tbx2) in Plp1-TAT mice subject to different treatments and analysed at distinct ages, as illustrated on the right. Orange arrows in (C)–(C’’’): one IBC/IPh with high Tdtomato and Tbx2 expression but weak Atoh1 expression. Orange arrows in (D)–(D’’’): one IBC/IPh with high Tdtomato and Tbx2 and also high Atoh1 expression, because Atoh1 is stabilized here by TMP treatment. Atoh1 expression is reversed to a weak level in Tdtomato+/Tbx2+ cells [orange arrows in (E)–(E’’’)] if mice are analysed at P7 (3 days after TMP treatment), but high expression can be restored in Tdtomato+/Tbx2+ cells [orange arrows in (F)–(F’’’)] if a third TMP treatment is administered 3 h before analysis at P7. (G)–(G’’’) Triple labeling for Myo7a, Tdtomato and Sox2 in cochleae of Plp1-TAT mice at P42. Arrows: one Tdtomato+/Sox2+ cell that does not express Myo7a and belongs to the IBCs/IPhs failing to become HCs. Scale bar: 20 μm (A’’’), (F’’’) and (G’’’).

The functional importance of Tbx2 in IHC development observed here prompted us to further hypothesize that Tbx2 together with Atoh1 should be capable of converting cochlear IBCs/IPhs, which localize near IHCs, into more differentiated new IHCs (i.e. into vGlut3+ cells) than what was obtained with Atoh1 alone in our previous study [26]. The IBCs/IPhs were effectively targeted using Plp1-CreER+ (Fig. 5A–A’’’), as reported in other studies [26–28]. Moreover, we aimed to only induce temporal Atoh1 expression, mimicking the Atoh1 expression observed during endogenous IHC development [5,29–31], and thus used the approach detailed below.

When the destabilizing domains (DDs) derived from *Escherichia coli* dihydrofolate reductase (DHFR) are fused with a protein of interest, rapid proteasomal degradation of the protein is triggered [32]; however, the cell-permeable small molecule trimethoprim (TMP) can bind to and stabilize the DDs, and thus TMP treatment results in the protein degradation being prevented in a rapid, reversible and TMP dose-dependent manner [32]. Furthermore, fusion of DHFR DDs to both N- and C-termini of a protein yields superior temporal control than does fusion to one terminus alone [33]. Therefore, for our analyses, we constructed a new strain, *Rosa26*-CAG-Loxp-Stop-Loxp-Tbx2*3 × V5-P2A-DHFR-Atoh1*3 × HA-DHFR-T2A-Tdtomato*/+* (abbreviated as *Rosa26*-LSL-TAT/+) (Fig. 5B and Supplementary Fig. S6A–F), in which Atoh1 protein was tagged with HA and DHFR; here, as expected, Atoh1 is unstable and undergoes rapid degradation under control conditions (Supplementary Fig. S6G) but becomes temporally stable in the presence of TMP (Supplementary Fig. S6H) [32,34]. Moreover, upon Cre-mediated recombination, Atoh1 (also tagged with three HA fragments), Tbx2 (tagged with three V5 fragments) and Tdtomato are transcribed from the same polycistronic mRNA in this strain.

We confirmed the transient and persistent expression of Atoh1 and Tbx2, respectively, in our model by using the four assays described here; in all cases, TMX was administered at P0 and P1 and TMP at P3 and P4, unless specified otherwise. First, in Plp1-CreER+; *Rosa26*-LSL-TAT/+ (abbreviated as Plp1-TAT) mice in the absence of TMX administration, no Tdtomato+, V5 (Tbx2)+ or HA (Atoh1)+ cells were observed. Second, Plp1-TAT mice that were administered TMX were divided into two groups—No-TMP and TMP-treated—and both groups were analysed at P4 (3 h after last TMP treatment). In cochleae from no-TMP mice, we detected Tdtomato+ cells in which V5 (Tbx2) expression was high but HA (Atoh1) expression was faint or undetectable (arrows in Fig. 5C–C’’’); by contrast, in TMP-treated mice, Tdtomato+ cells expressing high levels of Tbx2 and Atoh1 were present (arrows in Fig. 5D–D’’’). The faint Atoh1 expression in Fig. 5C might be due to occasional incomplete degradation of Atoh1 protein. Third, when we examined the TMP-treated mice at P7, we found that high Tbx2 and Tdtomato expression was maintained but Atoh1 expression was reversed to a faint or undetectable level in the same cells (arrows in Fig. 5E–E’’’). Fourth, Atoh1 expression was restored to a high level if an additional (third) dose of TMP was administered 3 h before sacrifice at P7 (arrows in Fig. 5F–F’’’). Collectively, these results supported our conclusions that whereas the expression of Tbx2 and Tdtomato solely relied on TMX, Atoh1 expression depended on both TMX and TMP, and that the Atoh1 protein level was reversible and TMP treatment stabilized Atoh1 only transiently (for <3 days).

### Transient Atoh1 and permanent Tbx2 expression together successfully convert neonatal IBCs/IPhs into vGlut3+ new IHCs

**Figure 6.**
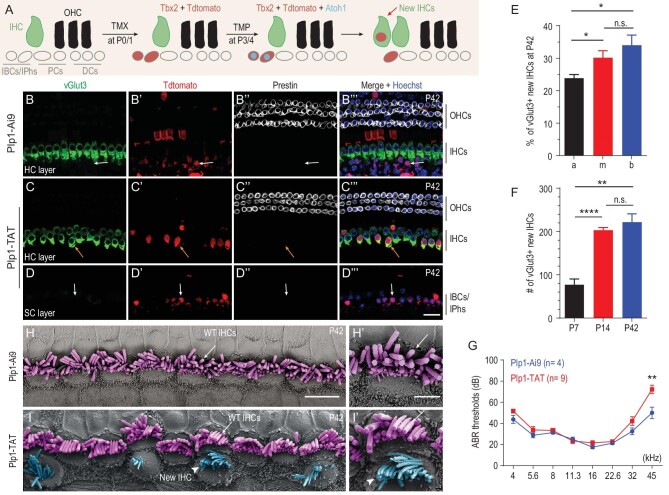
Transient Atoh1 and permanent Tbx2 expression convert neonatal IBCs/IPhs into vGlut3+ new IHCs. (A) Simple cartoon illustrating key cellular events in reprogramming process. (B)–(D’’’) Triple labeling for IHC marker vGlut3, Tdtomato and OHC marker Prestin in control Plp1-Ai9 (B)–(B’’’) and experimental Plp1-TAT (C)–(D’’’) mice at P42. Images show visualization at HC layer (B)–(C’’’) or SC layer (D)–(D’’’). Arrows in (B)–(B’’’): one Tdtomato+ IBC/IPh that expresses neither vGlut3 nor Prestin. Arrows in (C)–(C’’’): one new IHC that is Tdtomato+/vGlut3+/Prestin–. Arrows in (D)–(D’’’): one Tdtomato+ IBC/IPh that fails to undergo cell-fate change and expresses neither vGlut3 nor Prestin. (E) Percentage of vGlut3+ new IHCs in three cochlear turns, basal (b), middle (m) and apical (a), of Plp1-TAT mice at P42. Data are presented as means ± SEM (*n* = 5). Percentage of new IHCs in apical turn is lower than in middle and basal turns (*P < 0.05). (F) Total numbers of vGlut3+ new IHCs in entire cochleae of Plp1-TAT mice at P7 (*n* = 3), P14 (*n* = 5) and P42 (*n* = 5). Data are presented as means ± SEM. **P < 0.01; ****P < 0.0001. (G) ABR measurements between Plp1-Ai9 (blue line) and Plp1-TAT (red line) mice at P42. No significant difference except at 45 kHz (**P < 0.01). (H)–(I’) SEM analysis of cochlear samples from control Plp1-Ai9 mice (H)–(H’) and experimental Plp1-TAT mice (I)–(I’). White arrows in (H)–(H’) and (I)–(I’): same endogenous IHCs; white arrowheads in (I)–(I’): same new IHCs. Scale bars: 2 μm (H’); 5 μm (H); 20 μm (D’’’).

We first confirmed that Tbx2 alone cannot convert neonatal IBCs/IPhs into IHCs or general HCs by characterizing Plp1-TAT mice that were administered only TMX and then analysed at P42. All Tdtomato+ cells, which were derived from IBCs/IPhs, failed to express Myo7a (Fig. 5G–G’’’). We also did not observe Tdtomato+/vGlut3+ cells. Second, we ascertained whether simultaneously high levels of Atoh1 and Tbx2 can convert neonatal IBCs/IPhs into vGlut3+ new IHCs (Fig. 6A). In the cochleae of control Plp1-Ai9 mice at P42 (*n* = 3), neither the IHC marker vGlut3 nor the OHC marker Prestin was expressed in the Tdtomato+ cells that were IBCs/IPhs (arrows in Fig. 6B–B’’’). By contrast, Tdtomato+ cells expressing vGlut3, but not Prestin, were observed in the cochleae of Plp1-TAT mice at P42 (arrows in Fig. 6C–C’’’). Because these Tdtomato+/vGlut3+ cells were derived from IBCs/IPhs, they were defined as new IHCs (or conservatively named as IHC-like cells) and the new IHCs were found to be primarily adjacent to the endogenous IHCs that were vGlut3+/Tdtomato–. Among the 563 new IHCs identified in the middle and apical turns, 561 (99.6%) were located at the HC layer and likely to lose contact with the basement membrane (Supplementary Fig. S7A–A’’’ and Supplementary Video S1); by contrast, only 2/563 cells (0.4%) appeared to maintain contact with the basement membrane (Supplementary Fig. S7B–B’’’). Below and adjacent to these new IHCs were Tdtomato+ cells expressing neither vGlut3 nor Prestin (arrows in Fig. 6D–D’’’), which we defined as IBCs/IPhs that failed to become IHCs and were primarily located in the SC layer.

Next, we quantified the Tdtomato+/vGlut3+ new IHCs localized close to endogenous IHCs. Per 200-μm of cochlear duct (*n* = 5), 8.0 ± 0.8, 9.3 ± 0.7 and 5.2 ± 0.8 new IHCs were present in basal, middle and apical turns, respectively. Furthermore, we calculated the cell-fate conversion rate by normalizing the number of new IHCs to the total number of Tdtomato+ cells that were close to the endogenous IHCs within the same region: 34.0% ± 3.1%, 30.2% ± 2.2% and 23.9% ± 1.1% of the Tdtomato+ IBCs/IPhs were respectively converted into vGlut3+ new IHCs in the basal, middle and apical turns (Fig. 6E). However, the average cell-fate conversion rate differed by <1.5-fold among the three turns and thus, to simplify the subsequent analysis, we grouped the three turns together. Our results revealed 221.8 ± 19.1 new IHCs in the entire cochleae of Plp1-TAT mice at P42 (Fig. 6F), with an average cell-fate conversion rate of 29.5% ± 1.2%; the remaining ∼70.5% of the cells were IBCs/IPhs that failed to become new IHCs. Notably, the vGlut3+ new IHCs appeared at P7, but their numbers at P7 were significantly lower than at P14 and P42 (Fig. 6F). All the vGlut3+ new IHCs also expressed another IHC marker, Otoferlin, in Plp1-TAT mice, but not in Plp1-Ai9 mice, at P42 (Supplementary Fig. S8A–B’’’). Furthermore, when Myo7a was used as a marker to define new HCs in general, 239.3 ± 17.2 Tdtomato+/Myo7a+ new HCs were detected in the entire cochleae of Plp1-TAT mice at P42, but these cells were not present in Plp1-Ai9 mice (arrows in Supplementary Fig. S8C–D’’). Given the similar numbers (Supplementary Fig. S8E) and percentages (Supplementary Fig. S8F) of vGlut3+ new IHCs and Myo7a+ new HCs, we deduced that most, if not all, of the Myo7a+ new HCs adopted the IHC fate.

Lastly, auditory brainstem response (ABR) assay showed that hearing thresholds did not differ significantly between Plp1-Ai9 and Plp1-TAT mice at P42, except at 45 kHz (Fig. 6G). This result suggested that, except at high frequency (45 kHz), the additional IHCs did not affect the function of endogenous IHCs and this agreed with the finding in *Huwe1* mutant mice, which also harbor extra IHCs but display normal hearing ability [35]. Moreover, scanning electron microscopy (SEM) analysis at P42 revealed that, relative to control Plp1-Ai9 mice that contained a single row of endogenous IHCs (purple color in Fig. 6H and H’), Plp1-TAT mice harbored an additional but discontinuous row of IHCs (blue color) whose stereocilia showed a ‘bird-wing’ pattern (Fig. 6I and I’); 12.1 ± 1.2 (*n* = 3) extra IHCs with stereocilia were counted per 200 μm and 68.1% ± 4.8% of these cells featured relatively well organized stereocilia (white arrowheads in Fig. 6I). The numbers were higher than those determined through immunostaining analysis because we used SEM to scan areas with larger numbers of new IHCs. Nonetheless, compared to the endogenous IHCs, the stereocilia in these new IHCs were immature, variable in numbers and of a poorer quality. Considering these results collectively, we conclude that transient Atoh1 and permanent Tbx2 expression reprogrammed neonatal IBCs/IPhs into new IHCs that expressed the early pan-HC marker Myo7a, and the IHC-specific markers vGlut3 and Otoferlin, and possessed IHC-like stereocilia.

## DISCUSSION

The proliferation of undifferentiated cochlear sensory progenitors continues until E14.5 and is followed by a differentiation wave that moves in a basal-to-apical gradient [36,37]. Atoh1 is essential for specifying the general HC fate, because both IHCs and OHCs are lost in *Atoh1*^–/^^–^ mice [4] and Insm1 and Ikzf2 have recently been reported to be critical for OHC development [16,17]. However, the gene specifically necessary for IHC development has remained unknown. Our study has revealed that Tbx2 plays an essential role in maintaining the cell fate of differentiating and mature IHCs by preventing these cells from transdifferentiating into OHCs. Approximately 56.3% of OHC genes, including *Slc26a5* and *Ikzf2*, are upregulated and 26.7% of IHC genes, including *Slc17a8* and *Otof*, are downregulated in the *Tbx2^–/–^* IHCs that we defined here as iOHCs at P14. We also confirmed that Ikzf2 protein is highly expressed in these iOHCs (Fig. 3), further indicating that ectopic Ikzf2 expression by itself may be sufficient to transdifferentiate IHCs to OHCs [16,19]. Thus, we speculate that Tbx2 stabilizes the IHC fate partially by repressing *Ikzf2* expression. However, we cannot exclude the possibility that this is a secondary effect following cell-fate changes. Whether Tbx2 directly binds to *Ikzf2 cis*-regulatory elements warrants future investigation by using *in vivo* Tbx2 CUT&RUN assays in cochlear tissues.

The non-sensory SCs including IBCs/IPhs that localize in the medial cochlear portion and close to IHCs are plastic and can replenish themselves after damage in neonatal cochleae [28,38]. Determining how functional IHCs can be regenerated from these cochlear non-sensory SCs is a long-term goal in the inner-ear biology field. New IHCs that were previously derived from neonatal IBCs/IPhs through Atoh1 ectopic expression alone express the nascent HC marker Myo6 or Myo7a, but fail to turn on the expression of the late IHC marker vGlut3 [26]. We propose two possible interpretations: (i) Atoh1 is transiently expressed in WT IHCs but is persistently induced in the immature new IHCs [26]; and (ii) other key genes are required to further drive the differentiation of the new IHCs. In this study, we designed a new genetic model that enables transient Atoh1 and persistent Tbx2 expression to be effectively induced in neonatal IBCs/IPhs. Excitingly, we found that the differentiation status of the new IHCs is considerably more advanced than what was previously reported [26]. Furthermore, the reprogramming efficiency achieved using Atoh1 and Tbx2 was 29.5%, which is higher than the 17.8% measured with Atoh1 alone [26]. Thus, we propose that synergistic interactions exist between Atoh1 and Tbx2 during the cell-fate conversion process, much the same as between Atoh1 and Ikzf2 described in our OHC regeneration study [19] or between Atoh1 and Pou4f3 during endogenous HC development [39]. However, it remains unclear why the neonatal IBCs/IPhs that express endogenous Tbx2 fail to become vGlut3+ new IHCs when ectopic Atoh1 is induced [26]. One possibility is that Tbx2 exerts a dose-dependent effect on cell-fate determination. Moreover, the precise roles played by Tbx2 in the medial non-sensory SCs including IBCs/IPhs remain unknown.

While we were in the process of submitting this manuscript, a similar *Tbx2* conditional loss-of-function study in IHCs was reported in which *Tbx2* was deleted in either embryonic or neonatal differentiating IHCs [40]. Our present data confirm that Tbx2 is required for preventing differentiating IHCs from transdifferentiation into OHCs. Moreover, we extended *Tbx2* ablation in IHCs to P60/P61 and showed that Tbx2 also plays an essential role in stabilizing or maintaining the cell fate of fully mature IHCs. Notably, we also extended the characterization of iOHCs by performing single-cell transcriptomic analysis (Fig. 3), which enabled us to obtain a global gene-expression profile of the iOHCs. Because only 26.7% of the IHC genes were significantly decreased in iOHCs, our data raise the possibility that iOHCs and endogenous OHCs are not as similar as previously suggested, based on immunostaining with a couple of known IHC and OHC markers [40]. Nonetheless, both studies clearly support the notion that iOHCs resemble endogenous OHCs in several aspects. Lastly, our study showed that transient Atoh1 and permanent Tbx2 expression can convert neonatal IBCs/IPhs into vGlut3+ IHCs. Importantly, besides targeting cochlear IBCs/IPhs, Plp1-CreER+ also targets vestibular SCs and inner-ear glial cells [23,41]. Thus, a better option would be the future development of a new Cre or CreER mouse strain specific to IBCs/IPhs. The effect of damaging the endogenous IHCs on the transdifferentiating IBCs/IPhs into IHCs also warrants future investigation. In summary, the key findings in both studies will facilitate future treatment of IHC degeneration-associated hearing loss in the clinic.

## MATERIALS AND METHODS

### Mouse models

The mouse strains Plp1-CreER+ (Stock# 005 975) and *Rosa26*-LSL-Tdtomato/+ (Ai9; Stock# 007 909) were from The Jackson Laboratory. *Slc17a8^iCreER/+^*, which is also known as *vGlut3-P2A-iCreER/+*, was reported and described in detail in our previous study [18]. *Tbx2*-HA/+, *Tbx2^+/–^, Tbx2^flox/+^, Ikzf2^V5/+^* and *Rosa26*-LSL-TAT/+ mouse strains were generated by CRISPR/Cas9-mediated homologous recombination in one-cell-stage mouse zygotes. The PCR primers used for genotyping each strain and their amplicon sizes are described in Supplementary Table S3. All mice were raised in SPF-level animal rooms and animal procedures were performed according to the guidelines (NA-032-2019) of the IACUC of the Institute of Neuroscience (ION), CAS Center for Excellence in Brain Science and Intelligence Technology, Chinese Academy of Sciences.

### Sample processing, immunofluorescence assays and quantification

The dissected cochlear ducts were divided into three pieces: basal, middle and apical turns. The detailed immunostaining protocols have been described previously [42]. The iOHC percentage was calculated by normalizing the numbers of all iOHCs to the total number of IHCs. The 16-kHz frequency region was selected to quantify the numbers of Ctbp2+ puncta. The percentages of vGlut3+ new IHCs or Myo7a+ nascent HCs were calculated by normalizing the numbers of vGlut3+/Tdtomato+ or Myo7a+/Tdtomato+ cells to the total Tdtomato+ cells (but only those close to IHCs). Statistical analyses were performed using one-way ANOVA and Student’s *t*-test with Bonferroni corrections.

### ABR measurement, SEM preparation and analysis

ABR measurements were performed at 4, 5.6, 8, 11.3, 16, 22.6, 32 and 45 kHz on P42 mice, following our previously published protocol [18]. Student's *t*-tests were used to determine the statistical significance of differences in hearing thresholds at the same frequency among distinct mice (Figs 2I and 6G). For SEM, we used the protocol described in detail in our previous study [19].

### Preparation of cell suspensions, smart-seq single-cell RNA-seq and bioinformatics analysis

Cochlear samples were dissected out from Slc17a8-Ai9 mice at P14 or P30 and from Slc17a8-Tbx2cko-Ai9 mice at P14. The dissociated Tdtomato+ cells were manually picked. The picked endogenous IHCs and iOHCs were immediately subject to reverse-transcription and cDNA amplification by using a Smart-Seq HT kit (Cat# 634 437, Takara). The final libraries were subject to paired-end sequencing, which yielded ∼4 G of raw data per library. The FASTQ files of the smart-seq data were aligned to the mouse genome (GRCm38 mm10) by using Hisat2 alignment package (v2.1.0) [43]. Raw count matrices were generated using HTseq (v0.10.0) [44] and the TPM values were calculated using StringTie (v1.3.5) [45]. Trajectory analysis was performed in Monocle (v2.14.0) [46]. All the raw data of our single-cell RNA-seq analyses have been deposited in the GEO (Gene Expression Omnibus) under accession number: GSE199369.

## Supplementary Material

nwac156_Supplemental_Files
