# Supplementary material for: Development and transdifferentiation into inner hair cells require Tbx2
Source: Natl Sci Rev. 2022 Aug 9;9(12):nwac156. doi: 10.1093/nsr/nwac156 (PMC9844247; doi:10.1093/nsr/nwac156)

Supplemental Figure 5

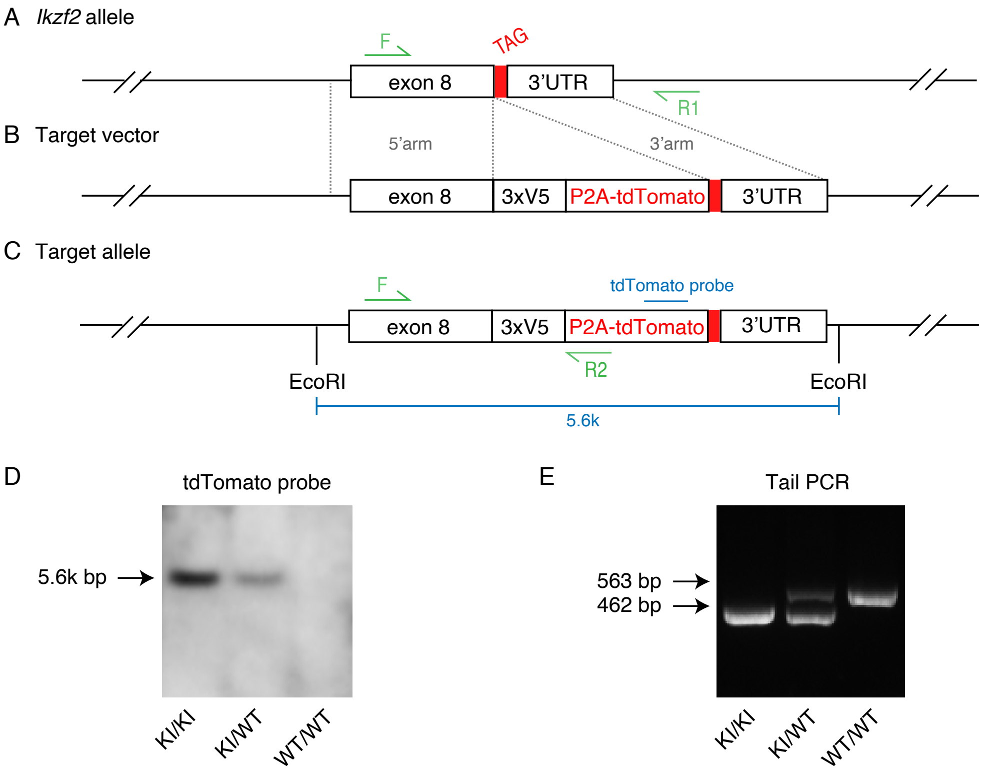

Supplement: nwac156_Supplemental_Files [file nwac156_supplemental_files.zip › Supplemental_Figure_5.pdf]
