# Supplementary material for: Development and transdifferentiation into inner hair cells require Tbx2
Source: Natl Sci Rev. 2022 Aug 9;9(12):nwac156. doi: 10.1093/nsr/nwac156 (PMC9844247; doi:10.1093/nsr/nwac156)
Supplement: nwac156_Supplemental_Files [file nwac156_supplemental_files.zip › Supplemental_Materials.docx]

Supplementary data for

**Development and transdifferentiation into inner hair cells require Tbx2**

Zhenghong Bi^1, 4^, Xiang Li^1, 4^, Minhui Ren^1, 2, 4^, Yunpeng Gu^1, 2^, Tong Zhu^1^, Shuting Li^1, 2^, Guangqin Wang^1, 2^, Suhong Sun^1, 2^, Yuwei Sun^1, 2^, Zhiyong Liu^1, 2, 3^ *

1. Institute of Neuroscience, State Key Laboratory of Neuroscience, CAS Center for Excellence in Brain Science and Intelligence Technology, Chinese Academy of Sciences; Shanghai, 200031, China.
2. University of Chinese Academy of Sciences; Beijing, 100049, China.
3. Shanghai Center for Brain Science and Brain-Inspired Intelligence Technology; Shanghai, 201210, China.

^4^ These authors contributed equally to this work.

^*^ Corresponding author. Email: [Zhiyongliu@ion.ac.cn](mailto:Zhiyongliu@ion.ac.cn)

**This PDF file includes:**

Supplemental Materials and Methods

Legends for Supplemental Figures 1 to 8

Legend for Supplemental Video 1

Supplemental References

**Supplemental Materials and Methods**

**Mouse models**

The mouse strains Plp1-CreER+ (Stock# 005975) and *Rosa26*-LSL-Tdtomato/+ (Ai9; Stock# 007909) were from The Jackson Laboratory. Slc17a8-P2A-iCreER/+, which is also known as *vGlut3-P2A-iCreER/+*, was reported and described in detail in our previous study (1). Both male and female mice were used. TMX (Cat# T5648, Sigma-Aldrich) was dissolved in corn oil (Cat# C8267, Sigma-Aldrich) and administrated at 3 mg/40 g body weight. TMP (Cat# T0667, Sigma-Aldrich) was dissolved in 1× phosphate-buffered saline (PBS) and administered at 300 μg/g body weight. All mice were bred and raised in SPF-level animal rooms, and animal procedures were performed according to the guidelines (NA-032-2019) of the IACUC of the Institute of Neuroscience (ION), CAS Center for Excellence in Brain Science and Intelligence Technology, Chinese Academy of Sciences.

**Generation of *Tbx2*-HA/+, *Tbx2^+/-^, Tbx2^flox/+^*, Ikzf2^V5/+^, and *Rosa26*-LSL-TAT/+ mouse strains**

The mouse strains described here were produced using the CRISPR/Cas9 approach. First, Tbx2-HA+ was constructed through CRISPR/Cas9-mediated homologous recombination in one-cell-stage mouse zygotes (Supplemental Figure 1); the sgRNA, close to the TGA stop codon, was 5ʹ-GACCCCCGACCTGCCACGCG -3ʹ. Second, Tbx2^+/-^ was generated by injecting two pre-tested efficient sgRNAs, sgRNA-1 and -2 (Supplemental Figure 2A), together with Cas9 mRNA; the two sgRNAs were 5ʹ- TGACCCGCCGTAAGGGCCTG-3ʹ and 5ʹ-AGGCTCCGAGGCGCCGACGT-3ʹ, respectively. Third, *Tbx2^flox/+^* was produced by co-injecting one-cell-stage mouse zygotes with two sgRNAs on the left and right sides of Tbx2 exon2, Cas9 mRNA, and the targeting vector (Supplemental Figure 2E); the two sgRNAs (Supplemental Figure 2D) were 5ʹ-ATGTCATGTCATTGTCGGGA-3ʹ and 5ʹ-AGGGGCCCCCACAGTCGAGT-3ʹ, respectively. Fourth, Ikzf2^V5/+^ was generated as illustrated in Supplemental Figure 5 by using the following sgRNA (close to the TAG stop codon): 5ʹ-AGGGGAGCACACATTCCACT-3ʹ. Lastly, *Rosa26*-LSL-TAT/+ was constructed similarly, as described in Supplemental Figure 6, by using the following sgRNA in the Rosa26 locus: 5ʹ-actccagtctttctagaaga-3ʹ.

In the case of all aforementioned mouse strains, once the Founder 0 (F0) mice were born, they were screened using tail-DNA PCR. The F0 mice with potentially correct gene targeting were selected and crossed with C57BL/6 WT mice to produce germ-line-stable F1 mice, which were subject to a second round of tail-DNA PCR screening. Moreover, Southern blotting was performed on all strains except *Tbx2^+/-^*. Only the F1 mice without any random insertion of the targeting vectors were selected for further breeding. The PCR primers used for genotyping each strain and their amplicon sizes are described in Supplemental Table 3.

**Sample processing and immunofluorescence assays**

Mice were anesthetized and then perfused with fresh ice-cold 1×PBS and 4% paraformaldehyde (PFA) in 1×PBS (pH 7.4), after which inner ear samples were carefully dissected out and further fixed in 4% PFA solution on rotator overnight at 4°C. On the next day, the samples were washed thrice with 1×PBS, decalcified in 120 mM EDTA (Cat# ST066, Beyotime) in 1×PBS at room temperature for either 24 h in the case of mice aged between P7 and P14 or 48 h for mice older than P14, and then washed thrice more with 1×PBS before proceeding to the whole-mount dissection described below. Inner ear samples from mice younger than P7 were dissected without EDTA treatment.

The dissected cochlear ducts were divided into three pieces, comprising the basal, middle, and apical turns. Each turn was immunostained in parallel. Briefly, cochlear samples were first incubated in blocking solution containing 1% Triton X-100 (X100-500ML, Sigma) and 5% bovine serum albumin (BSA; Cat# BP1605, Fisher Scientific) and then incubated overnight (on a rotator) at 4°C with primary antibodies dissolved in a solution containing 0.1% Triton X-100 and 5% BSA. On the second day, the samples were washed thrice with 1×PBS containing 0.1% Triton X-100 and then incubated with appropriate secondary antibodies (diluted in the 0.1% Triton X-100 and 5% BSA solution).

The following primary antibodies were used in this study: anti-HA (rat, 1:200; 11867423001, Roche), anti-V5 (mouse, 1:500; MCA1360, Bio-Rad), anti-Prestin (goat, 1:1000; sc-22692, Santa Cruz), anti-vGlut3 (rabbit, 1:500; 135203, Synaptic Systems), anti-Otoferlin (mouse, 1:500; ab53233, Abcam), anti-Myo7a (rabbit, 1:500; 25-6790, Proteus Biosciences), anti-Ctbp2 (mouse, 1:200; 612044, BD Biosciences), anti-Sox2 (goat, 1:1000; sc-17320, Santa Cruz), anti-Myo6 (rabbit, 1:500; 25-6791, Proteus Biosciences), and anti-Slc7a14 (rabbit, 1:500; HPA045929, Sigma-Aldrich). Lastly, the samples were counterstained with Hoechst 33342 solution in PBST (1:1000; 62249, Thermo Scientific) to visualize nuclei, and were mounted using Prolong Gold antifade medium (P36930, Thermo Scientific). The detailed immunostaining protocols have been described previously (2). Nikon C2, TiE-A1 Plus, and NiE-A1 Plus (Nikon, Japan) confocal microscopes were used to capture images.

**Calculating percentages of iOHCs, ribbon synapses, and new IHCs**

The three pieces of the cochlear duct were first scanned under a 10× lens of the confocal microscope and the total length of the pieces was calculated by drawing a line between IHCs and OHCs by using ImageJ software. Next, each cochlea was divided into three portions of equal length and each region was assigned into the basal, middle, or apical turn. In the Tbx2 cko model, the numbers of total IHCs (or cell types derived from endogenous IHCs) were calculated by adding the iOHCs (Prestin^High^/vGlut3^Low^, Prestin^High^/Otoferlin^Low^, or Prestin^High^/Slc7a14^Low^) and the IHCs that failed to become iOHCs (Prestin^Low^/vGlut3^High^, Prestin^Low^/Otoferlin^High^, or Prestin^Low^/Slc7a14^High^). Entire cochlear turns were scanned under a 60× lens of the confocal microscope to quantify the percentages of iOHCs (Figures 2A-H, Supplemental Figure 3A-F, and Figure 4A-D) with minimal variation. The iOHC percentage was calculated by normalizing the numbers of all iOHCs to the total number of IHCs. To quantify the numbers of Ctbp2+ puncta, which represent ribbon synapses, we selected the 16 kHz frequency region based on the method mentioned previously (3) and performed z-stack scanning (with an 0.41 μm interval) on the cochlear samples (Supplemental Figure 3G-I).

For quantifying the numbers or percentages of vGlut3+/Tdtomato+ new IHCs (Figure 6C-F) or Myo7a+/Tdtomato+ nascent HCs (Supplemental Figure 8), three areas in each turn were scanned (60×) and the average number per mouse was obtained. The percentages of vGlut3+ new IHCs or Myo7a+ nascent HCs were calculated by normalizing the numbers of vGlut3+/Tdtomato+ or Myo7a+/Tdtomato+ cells to the total Tdtomato+ cells (but only those close to IHCs) that included new IHCs/HCs and the Tdtomato+ IBCs/IPhs that failed to become new IHCs or HCs. Notably, the total numbers of vGlut3+ new IHCs (Figure 6F) or Myo7a+ cells (Supplemental Figure 8E) in the entire cochlear duct were calculated by multiplying the numbers determined under the confocal microscope (60×) with ~5733.3 μm (total cochlear length)/~230.9 μm (length under 60× lens). Statistical analyses were performed using one-way ANOVA and Student’s *t* test with Bonferroni corrections.

**ABR measurement and SEM preparation and analysis**

ABR measurements were performed at 4, 5.6, 8, 11.3, 16, 22.6, 32, and 45 kHz on P42 mice, following our previously published protocol (1). Student’s *t* tests were used to determine the statistical significance of differences in hearing thresholds at the same frequency among distinct mice (Figures 2I and 6G). For SEM, we used the protocol described in detail in our previous study (4).

**Preparation of cell suspensions and smart-seq single-cell RNA-seq**

Cochlear samples were dissected out from Slc17a8-Ai9 mice at P14 or P30 and from Slc17a8-Tbx2cko-Ai9 mice at P14, and after removing neural regions to the extent possible, the cochlear sensory epithelium was incubated in a choline chloride solution containing 20 U/mL papain (Cat# LK003178, Worthington) and 100 U/mL DNase I (Cat# LK003172, Worthington) for 20 min at 37°C; following this incubation, the samples were digested with a protease (Cat# P5147, Sigma; 1 mg/mL) and dispase (Cat# LS02104, Worthington; 1 mg/mL) for 20 min at 25°C. The choline chloride solution contained 92 mM choline chloride, 2.5 mM KCl, 1.2 mM NaH_2_PO_4_, 30 mM NaHCO_3_, 20 mM HEPES, 25 mM glucose, 5 mM sodium ascorbate, 2 mM thiourea, 3 mM sodium pyruvate, 10 mM MgSO_4_.7H_2_O, 0.5 mM CaCl_2_.2H_2_O, and 12 mM N-acetyl-L-cysteine. The post-digested cochlear samples were next gently triturated using fire-polished glass Pasteur pipettes (13-678-20b, Fisher) with four distinct pore sizes; the pipette with the largest pore size was used first and that with the smallest pore size was used last.

Lastly, the dissociated Tdtomato+ cells were manually picked under a fluorescence dissection microscope (M205FA, Leica). Notably, in the Slc17a8-Tbx2cko-Ai9 model, we could distinguish the Tdtomato+ iOHCs (smaller size, picked) from the Tdtomato+ IHCs that did not undergo cell-fate change (larger size, not picked). The picked endogenous IHCs and iOHCs were immediately subject to reverse-transcription and cDNA amplification by using a Smart-Seq HT kit (Cat# 634437, Takara), and the post-amplified cDNA (1 ng) of each cell was processed for final library construction by using a TruePrep DNA Library Prep Kit V2 for Illumina (Cat# TD503-02, Vazyme) and a TruePrep Index Kit V2 for Illumina (Cat# TD202, Vazyme). The final libraries were subject to paired-end sequencing on the Illumina Novaseq platform, which yielded ~4 G of raw data per library.

**Bioinformatics analysis**

The FASTQ files of the smart-seq data were aligned to the mouse genome (GRCm38 mm10) by using Hisat2 alignment package (v2.1.0) (5). Raw count matrices were generated using HTseq (v0.10.0) (6), and the TPM values were calculated using StringTie (v1.3.5) (7). For integration analysis of 10× genomic and smart-seq data, normalization (NormalizeData), principal component analysis (RunPCA), dimensional reduction (RunUMAP), unsupervised clustering (FindNeighbors/FindClusters), and integration (FindIntegrationAnchors/IntegrateData) were performed using Seurat (v3.2.3) (8). The DEGs (p<0.05; absolute value of (log2 Fold-Change): >2) between P30_WT IHCs and P30_WT OHCs, as well as between P14_WT IHCs and P14_iOHCs, were determined using DESeq2 (v1.34.0) (9). Furthermore, the DEGs between P30_WT OHCs and P30_WT IHCs whose averaged TPM value was >16 were defined as OHC and IHC genes.

In the case of the 10× genomic single-cell data from the previous study (10), cells showing above-zero expression of *Insm1*, *Myo6*, and *Atoh1* were identified as E16_WT OHCs. Moreover, P1_WT OHCs were defined as cells with above-zero expression of *Bcl11b*, *Myo6*, *Myo7a*, and *Atoh1*, and P7_WT OHCs as cells with above-zero expression of *Slc26a5*, *Myo6*, *Ocm*, and *Ikzf2*. Similarly, E16_WT IHCs and P7_WT IHCs were defined as cells with above-zero expression of *Myo6* and *Fgf8*, and cells with above-zero expression of *Myo7a*, *Myo6*, and *Fgf8* were classified as P1_WT IHCs.

Trajectory analysis was performed in Monocle (v2.14.0) (11). The pre-processed matrix in the Seurat object (above) was used. The top 2000 most-variable genes assessed from the Seurat object (“FindVariableFeatures”) were used as input to simulate the pseudotime trajectory. Cells were fitted onto the backbone of the trajectory graph by using the “orderCells” function in Monocle. All the raw data of our single-cell RNA-seq analyses have been deposited in the GEO (Gene Expression Omnibus) under accession number GEO: GSE199369.

**Supplemental Figure Legends**

**Supplemental Figure 1.** **Tbx2 expression is turned on in early otocyst cells but is gradually depressed in cochlear lateral progenitors. (A-C)** Detailed illustration of design of Tbx2*3×HA-P2A-iCreER-T2A-EGFP/+ (*Tbx2*-HA/+). Immediately before the stop codon in *Tbx2* WT allele (A), the fragment containing 3×HA-P2A-iCreER-T2A-EGFP (B) is inserted, generating the final targeted *Tbx2* allele (C). **(D-E)** Southern blotting results obtained using both 3ʹ-probe (D) and internal iCre probe (E) confirm absence of random insertion of donor DNA (B). **(F)** Representative image of tail-DNA PCR used to distinguish knockin (KI) and WT alleles. **(G-G’’)** Dual staining of HA (Tbx2) and Sox2 in *Tbx2*-HA/+ embryos at E9.5. Tbx2 is highly expressed in otic vesicle (OV) cells. **(H-I’’)** Double staining of Myo6 and HA (Tbx2) in basal (H-H’’) and apical (I-I’’) turns of *Tbx2*-HA/+ cochlear samples at E15.5. Tbx2 expression is maintained in apical lateral progenitors (LPs) but is undetectable in basal LPs. IHC: inner hair cell; GER: greater epithelial ridge cells; LER: lesser epithelial ridge cells; LPs: lateral progenitors; MPs: medial progenitors; ov: otic vesicle; hb: hindbrain. Scale bar: 200 μm (G’’); 20 μm (H’’).

**Supplemental Figure 2. Generation of germ-line *Tbx2^+/-^* and conditional *Tbx2 ^flox/+^* strains. (A-C)** *Tbx2* exons and introns between sgRNA-1 and -2 (arrows in A) are deleted, as confirmed by Sanger sequencing (B). WT and Tbx2-null alleles yield tail-DNA PCR bands of 401 and 728 bp, respectively (C). **(D-F)** *Tbx2* WT allele (D) is recombined with the targeting vector in which exon 2 is flanked by two loxp sequences (E), producing the post-targeted floxed *Tbx2* allele (F). **(G-H)** Southern blotting results obtained using both internal probe (G) and 3ʹ-probe (H) show absence of random insertion of targeting vector. **(I)** Representative image of tail-DNA PCR used to identify WT and floxed *Tbx2* alleles.

**Supplemental Figure 3. Expression of** **IHC markers Otoferlin and Slc7a14 and number of ribbon synapses are decreased at P42 in Tbx2 absence. (A-D’’)** Double staining of IHC marker Otoferlin (A-B’’) or Slc7a14 (C-D’’) and Prestin in WT (A-A’’ and C-C’’) and Tbx2 cko (B-B’’ and D-D’’) cochlear samples. Yellow arrows: one Prestin^High^/Otoferlin^Low^ (B-B’’) and one Prestin^High^/Slc7a14^Low^ (D-D’’) iOHC; white asterisks: IHCs that fail to undergo cell-fate conversion and maintain expression of Otoferlin (B-B’’) and Slc7a14 (D-D’’), which are respectively included into Otoferlin^High^/Prestin^Low^ and Slc7a14^High^/Prestin^Low^ populations. **(E-F)** Quantification of Prestin^High^/vGlut3^Low^, Prestin^High^/Otoferlin^Low^, and Prestin^High^/Slc7a14^Low^ (E) cells and vGlut3^High^/Prestin^Low^, Otoferlin^High^/Prestin^Low^, and Slc7a14^High^/Prestin^Low^ (F) cells. No significant difference is detected. **(G-H’’)** Double staining of Slc7a14 and ribbon synapse marker Ctbp2 in WT (G-G’’) and Tbx2 cko (H-H’’) cochlear samples. White dotted circles: one endogenous IHC in WT cochlea (G-G’’) and one IHC that does not undergo cell-fate change and maintains Slc7a14 expression (H-H’’). Yellow dotted circles: one iOHC. **(I)** Quantification of synapse number per WT-IHC, iOHC, or WT-OHC, based on number of Ctbp2+ puncta. Data are presented as means ± SEM. ** p<0.01; **** p<0.0001. Synapse numbers in iOHCs are lower than in WT-IHCs but higher than in WT-OHCs. Scale bar: 20 μm (H’’).

**Supplemental Figure 4. Molecular features of P14_iOHCs. (A)** Volcano plot of all differentially expressed genes between P14_WT IHCs and P14_iOHCs. Volcano plot is generated based on gene-expression fold-change and p value of statistical difference between P14_WT IHCs and P14_iOHCs; however, absolute expression level of each gene is not considered. Red arrow: *Ikzf2*, a gene that is also highlighted in Figure 3C, where gene-expression level is considered. **(B-C)** Trajectory analysis of mixed cell populations including 8 cell types: E16_WT IHCs, E16_WT OHCs, P1_WT IHCs, P1_WT OHCs, P7_WT IHCs, P7_WT OHCs, P30_WT OHCs, and P14_iOHCs; these are plotted separately in (B) and together in (C). The combined plot in (C) is enlarged relative to each panel in (B) to improve visualization of the distribution of each cell type. Arrows in (C): calculated developmental direction.

**Supplemental Figure 5. Generation of *Ikzf2**3×V5-P2A-Tdtomato/+ (*Ikzf2*^V5/+^) mouse strain. (A-C)** Immediately before the TAG stop codon in WT *Ikzf2* allele (A), the fragment containing 3×V5-P2A-Tdtomato (B) is inserted, generating the post-targeted allele (C). **(D)** Southern blotting with internal Tdtomato probe yields a single 5.6k bp band, confirming absence of random insertion of targeting vector (B). **(E)** Representative image of tail-DNA PCR used to distinguish WT and KI alleles.

**Supplemental Figure 6. Construction of new *Rosa26-*LSL-TAT/+ strain. (A-C)** In WT *Rosa26* allele (A), we inserted a long polycistronic DNA element (B) containing Tbx2 fused with three V5 fragments at its C-terminus, Atoh1 fused with one DHFR element at its N-terminus and three HA fragments and another DHFR element at its C-terminus, and Tdtomato; the post-targeted allele is shown in (C). **(D-E)** Southern blotting results obtained with internal (D) and external 3ʹ (E) probes confirm absence of random insertion of targeting vector (B) in *Rosa26-*LSL-TAT/+ mouse genome. **(F)** Tail-DNA PCR readily distinguishes WT (469 bp) and KI (412 bp) alleles. **(G-H)** Schematic depiction of Atoh1-DHFR cassette stability relying on presence of TMP.

**Supplemental Figure 7. Majority of new IHCs are located in HC layer and appear to lose contact with basement membrane.** Triple labeling for vGlut3 (A and B), Prestin (A’ and B’), and Tdtomato (A’’ and B’’) in cochlear tissues of Plp1-TAT mice at P42. For each channel and the merged channel with Hoechst staining (A’’’ and B’’’), confocal scans in XY, YZ, and XZ axes are shown, as illustrated in (A’’’ and B’’’). Most of the new IHCs (99.6%) that express Tdtomato and vGlut3, but not Prestin, translocate to HC layer and are likely to lose contact with basement membrane. Yellow arrows in (A’’’): one such new IHC; white arrows in (B’’’): one new IHC that shows long basal protrusion and appears to maintain contact with basement membrane, and this type of new IHC represents the minority (only 0.4%). Scale bar: 20 μm (A’’’ and B’’’).

**Supplemental Figure 8. New IHCs also express another IHC marker Otoferlin, and pan-HC marker Myo7a. (A-B’’’)** Triple labeling for vGlut3, Tdtomato, and Otoferlin in cochleae of control Plp1-Ai9 (A-A’’’) and Plp1-TAT (B-B’’’) mice at P42; both were treated with TMX at P0 and P1 and TMP at P3 and P4. Arrows in (A-A’’’) and (B-B’’’): one IBC/IPh that is Tdtomato+/vGlut3-/Otoferlin- and one new IHC that is Tdtomato+/vGlut3+/Otoferlin+, respectively. **(C-D’’)** Double labeling for Myo7a and Tdtomato in cochleae of Plp1-Ai9 (C-C’’) and Plp1-TAT (D-D’’) mice. Arrows in (C-C”) and (D-D’’): one IBC/IPh that is Tdtomato+/Myo7a- and one new IHC that is Tdtomato+/Myo7a+, respectively. **(E-F)** Quantification of new IHC numbers (E) and percentages (F) among all Tdtomato+ cells, using either Myo7a or vGlut3 as a marker, at P42. Scale bar: 20 μm (B’’’ and D’’).

**Supplemental Video 1 Legend**

Confocal data presented in Supplemental Figure 7 were processed using Imaris software. Red, green, white and blue: Tdtomato, vGlut3, Prestin expression patterns and Hoechst staining, respectively. Yellow: vGlut3+/Tdtomato+/Prestin- cells, which are new IHCs located close to the endogenous IHCs (vGlut3+/Tdtomato-/Prestin-). Whereas new IHCs are translocated to HC layer and should lose contact with basement membrane, Tdtomato+ SCs that do not express vGlut3 are retained at SC layer.

**Supplemental References**

1. Li, C., Shu, Y., Wang, G., Zhang, H., Lu, Y., Li, X., Li, G., Song, L. and Liu, Z. (2018) Characterizing a novel vGlut3-P2A-iCreER knockin mouse strain in cochlea. *Hear Res*, **364**, 12-24.

2. Liu, Z., Owen, T., Zhang, L. and Zuo, J. (2010) Dynamic expression pattern of Sonic hedgehog in developing cochlear spiral ganglion neurons. *Dev Dyn*, **239**, 1674-1683.

3. Muller, M., von Hunerbein, K., Hoidis, S. and Smolders, J.W. (2005) A physiological place-frequency map of the cochlea in the CBA/J mouse. *Hear Res*, **202**, 63-73.

4. Sun, S., Li, S., Luo, Z., Ren, M., He, S., Wang, G. and Liu, Z. (2021) Dual expression of Atoh1 and Ikzf2 promotes transformation of adult cochlear supporting cells into outer hair cells. *Elife*, **10**.

5. Kim, D., Langmead, B. and Salzberg, S.L. (2015) HISAT: a fast spliced aligner with low memory requirements. *Nat Methods*, **12**, 357-360.

6. Anders, S., Pyl, P.T. and Huber, W. (2015) HTSeq--a Python framework to work with high-throughput sequencing data. *Bioinformatics*, **31**, 166-169.

7. Pertea, M., Pertea, G.M., Antonescu, C.M., Chang, T.C., Mendell, J.T. and Salzberg, S.L. (2015) StringTie enables improved reconstruction of a transcriptome from RNA-seq reads. *Nat Biotechnol*, **33**, 290-295.

8. Stuart, T., Butler, A., Hoffman, P., Hafemeister, C., Papalexi, E., Mauck, W.M., 3rd, Hao, Y., Stoeckius, M., Smibert, P. and Satija, R. (2019) Comprehensive Integration of Single-Cell Data. *Cell*, **177**, 1888-1902 e1821.

9. Love, M.I., Huber, W. and Anders, S. (2014) Moderated estimation of fold change and dispersion for RNA-seq data with DESeq2. *Genome Biol*, **15**, 550.

10. Kolla, L., Kelly, M.C., Mann, Z.F., Anaya-Rocha, A., Ellis, K., Lemons, A., Palermo, A.T., So, K.S., Mays, J.C., Orvis, J. *et al.* (2020) Characterization of the development of the mouse cochlear epithelium at the single cell level. *Nat Commun*, **11**, 2389.

11. Trapnell, C., Cacchiarelli, D., Grimsby, J., Pokharel, P., Li, S., Morse, M., Lennon, N.J., Livak, K.J., Mikkelsen, T.S. and Rinn, J.L. (2014) The dynamics and regulators of cell fate decisions are revealed by pseudotemporal ordering of single cells. *Nat Biotechnol*, **32**, 381-386.
