# Supplementary figures and images for: Development and transdifferentiation into inner hair cells require Tbx2
Source: Natl Sci Rev. 2022 Aug 9;9(12):nwac156. doi: 10.1093/nsr/nwac156 (PMC9844247; doi:10.1093/nsr/nwac156)

# Supplemental Figure 1

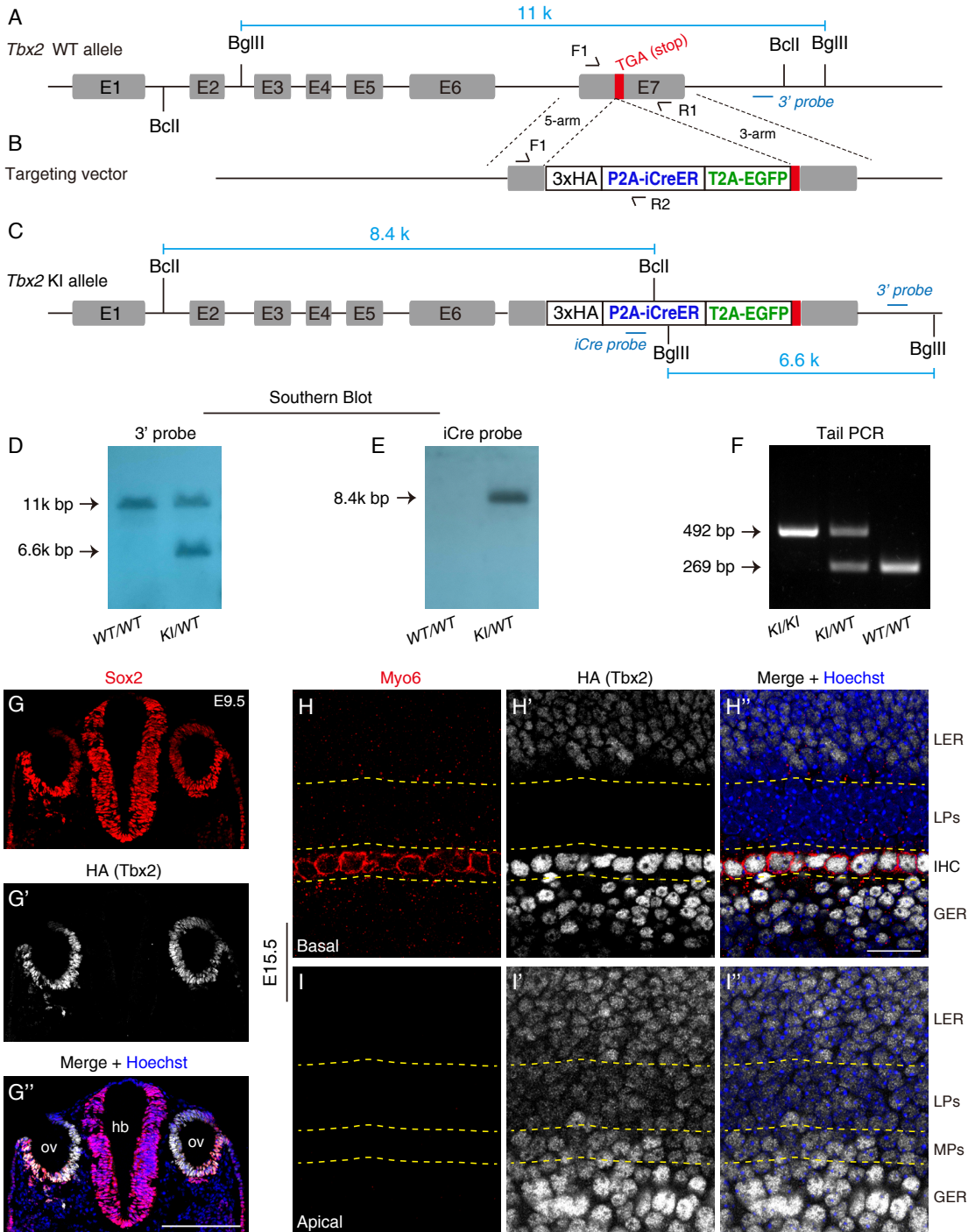

Supplement: nwac156_Supplemental_Files [file nwac156_supplemental_files.zip › Supplemental_Figure_1.pdf]

Supplemental Figure 2

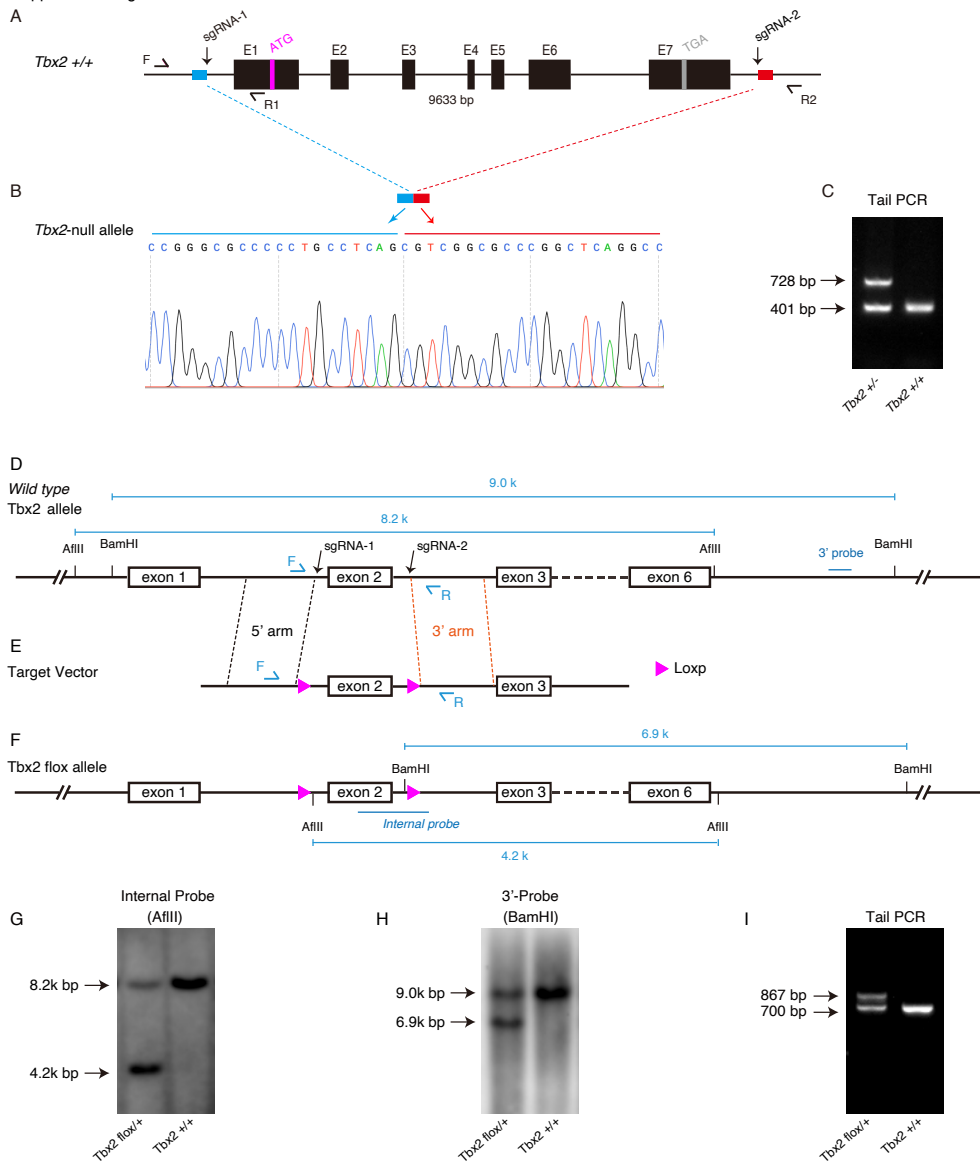

Supplement: nwac156_Supplemental_Files [file nwac156_supplemental_files.zip › Supplemental_Figure_2.pdf]

Supplemental Figure 3

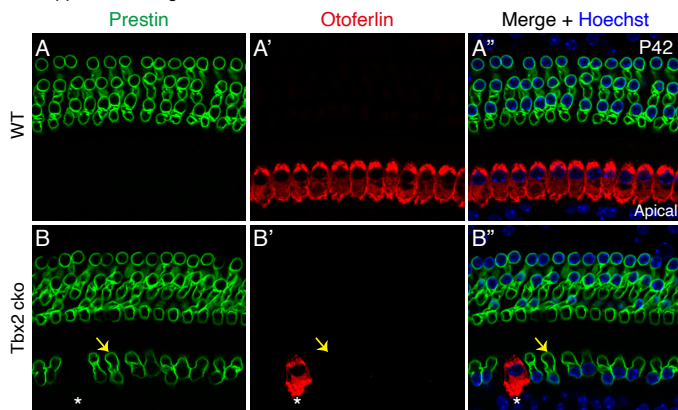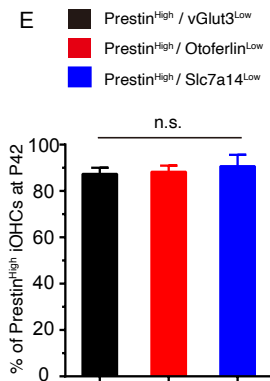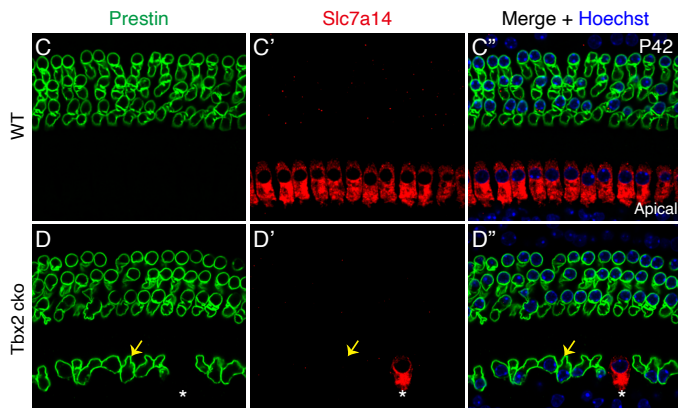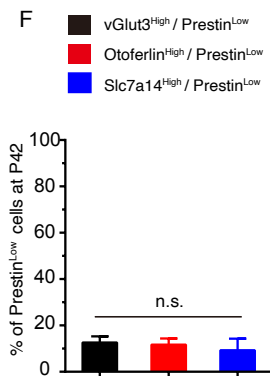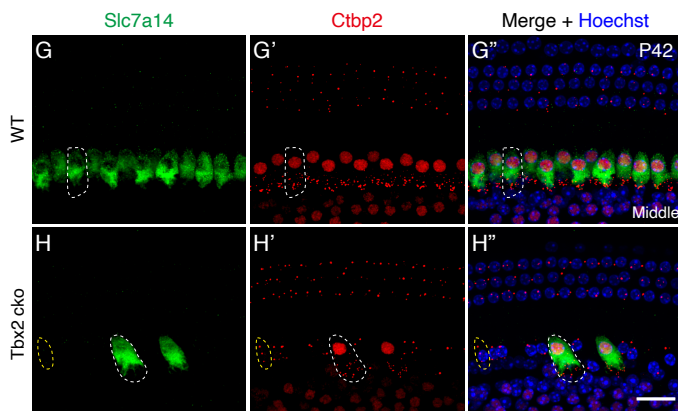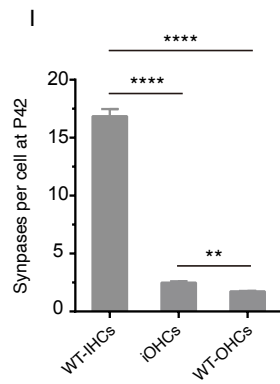

Supplement: nwac156_Supplemental_Files [file nwac156_supplemental_files.zip › Supplemental_Figure_3.pdf]

Supplemental Figure 4

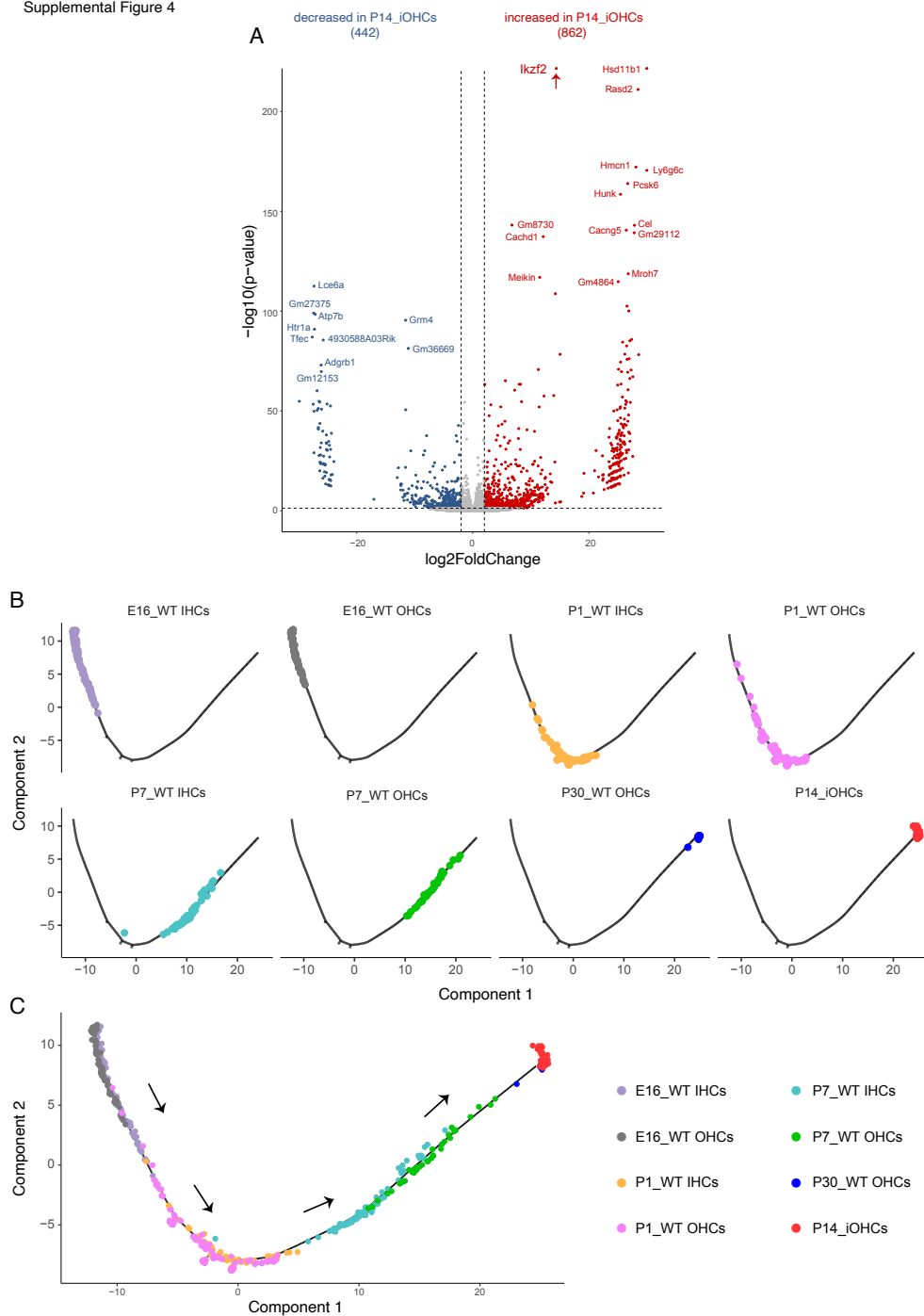

Supplement: nwac156_Supplemental_Files [file nwac156_supplemental_files.zip › Supplemental_Figure_4.pdf]

Supplemental Figure 6

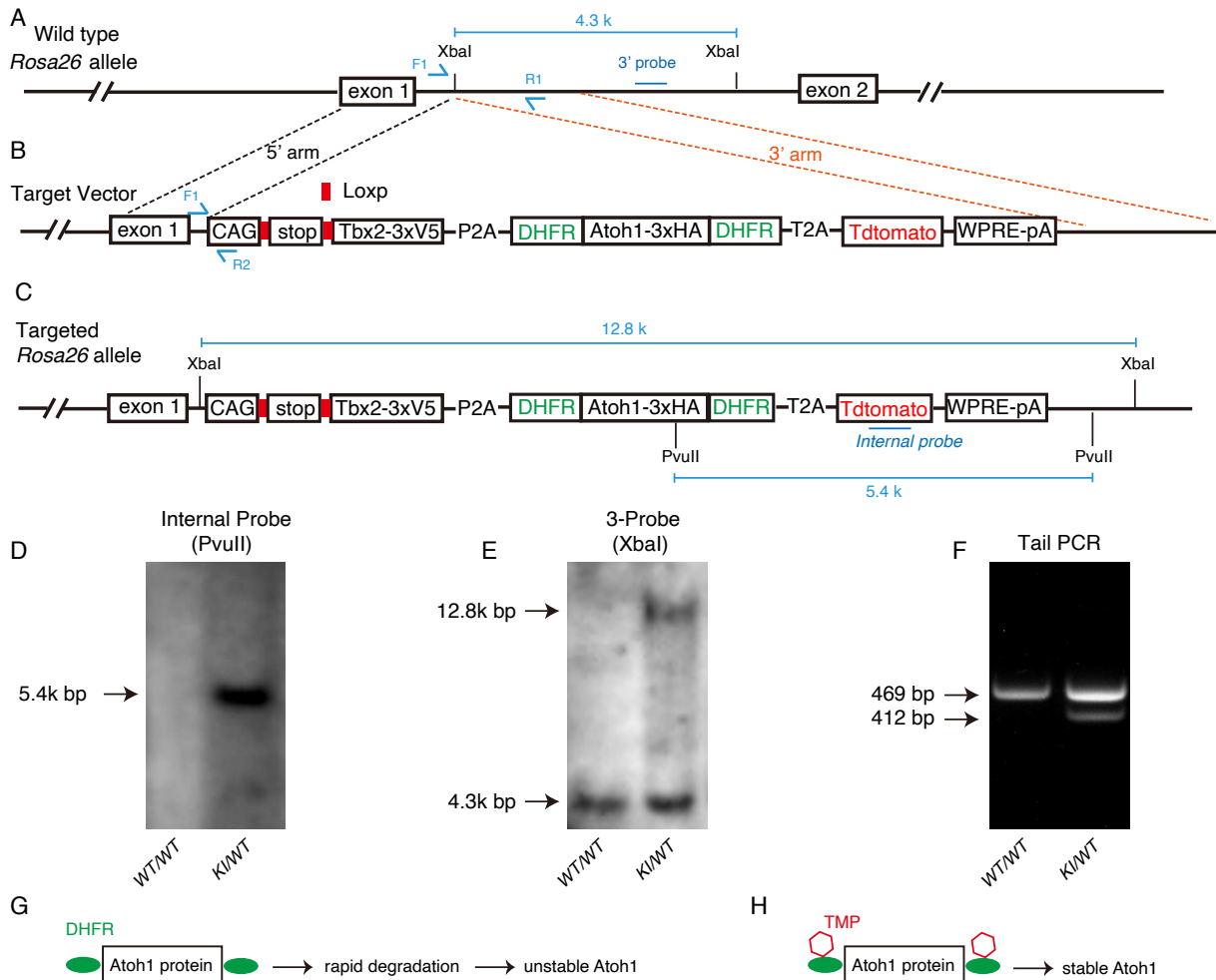

Supplement: nwac156_Supplemental_Files [file nwac156_supplemental_files.zip › Supplemental_Figure_6.pdf]

Supplemental Figure 7

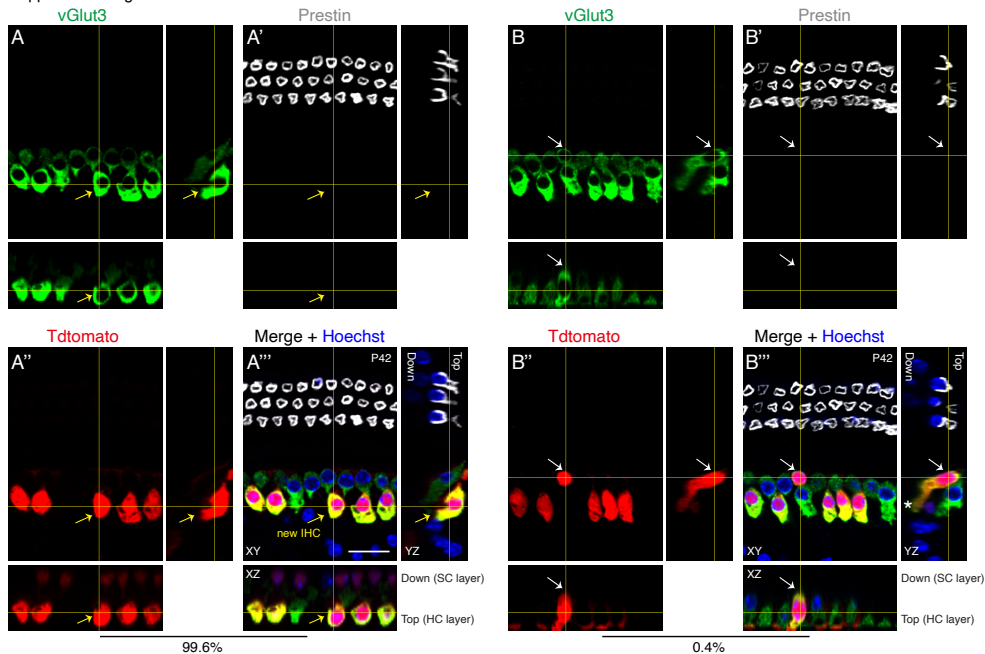

Supplement: nwac156_Supplemental_Files [file nwac156_supplemental_files.zip › Supplemental_Figure_7.pdf]

Supplemental Figure 8

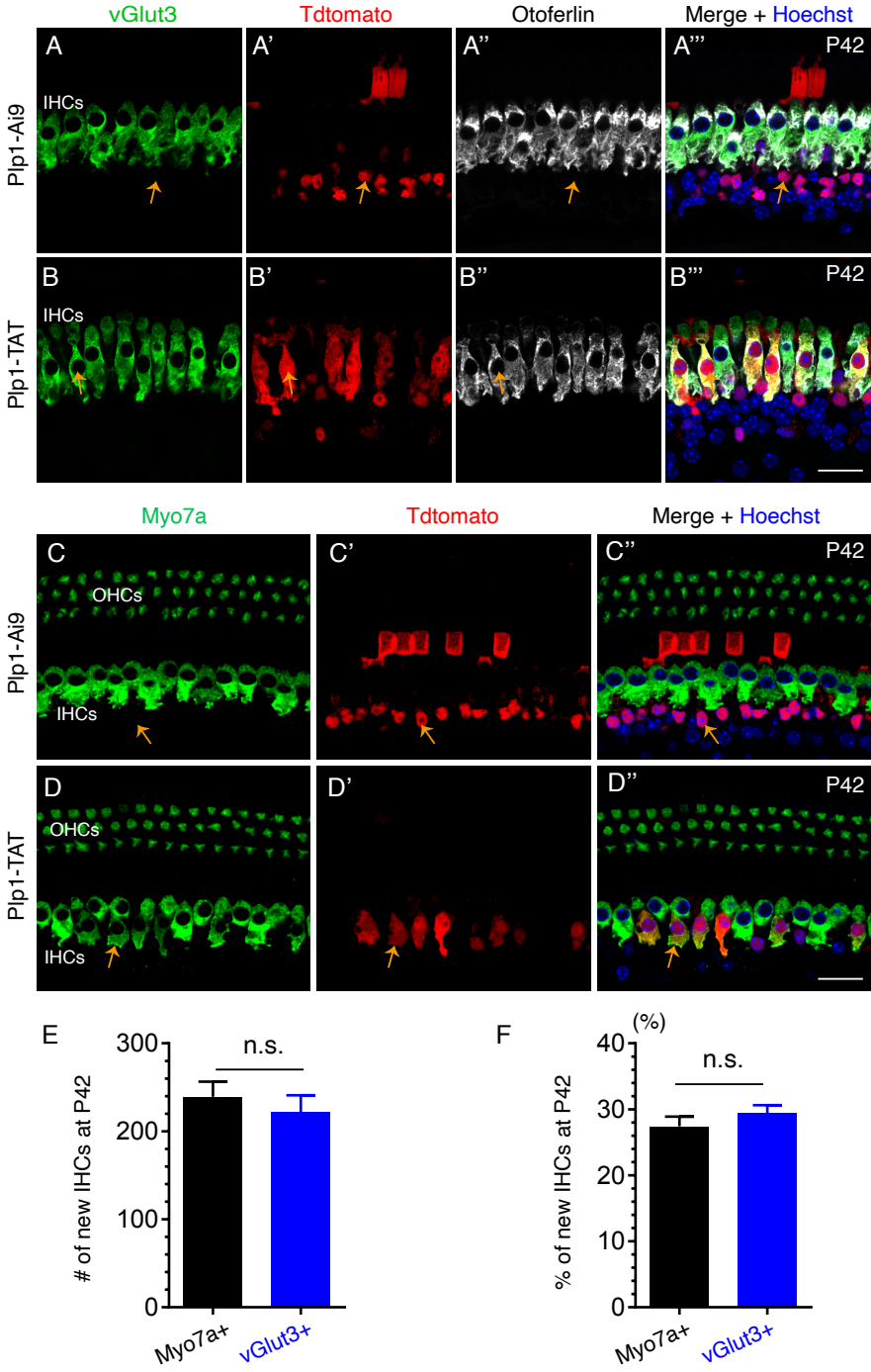

Supplement: nwac156_Supplemental_Files [file nwac156_supplemental_files.zip › Supplemental_Figure_8.pdf]
